# Revisiting the unobtrusive role of exogenous stem cells beyond neural circuits replacement in spinal cord injury repair

**DOI:** 10.7150/thno.103033

**Published:** 2025-01-02

**Authors:** Runlin Wen, Ge Long, Xinghui He, Kai Zhang, Wanrong Ma, Yeyu Shen, Zhifeng Xiao, Yannan Zhao, Dingyang Liu, Jianwu Dai, Xing Li

**Affiliations:** 1 Department of biochemistry and molecular biology, College of Life Sciences, Central South University, Changsha, 410078, Hunan, China.; 2 Department of Neurosurgery, Xiangya Hospital of Central South University, Changsha 410008, China.; 3 Department of Anesthesia, the Third Xiangya Hospital of Central South University, Changsha 410013, China.; 4 Center for Regenerative Medicine, State Key Laboratory of Molecular Developmental Biology, Institute of Genetics and Developmental Biology, Chinese Academy of Sciences, Beijing 100101, China.

**Keywords:** spinal cord injury, non-human primate, stem cell transplantation, immunosuppression, endogenous neuronal regeneration

## Abstract

**Rationale:** Stem cell transplantation is a promising strategy to establish neural relays in situ for spinal cord injury (SCI) repair. Recent research has reported short-term survival of exogenous cells, irrespective of immunosuppressive drugs (ISD), results in similar function recovery, though the mechanisms remain unclear. This study aims to validate this short-term repair effect and the potential mechanisms in large animals.

**Methods:** In this study, human spinal cord neural progenitor cells (hscNPCs) and human umbilical cord mesenchymal stem cells (hUMSCs) were transplanted into two different SCI model without ISD, respectively; Immunofluorescence was utilized to visualize neuronal regeneration and angiogenesis in the lesion site. Motor evoked potentials (MEPs) were detected to assess the integrity of motor pathways. And RNA sequencing was used to observe transcriptomic changes at the edge of the lesion.

**Results:** The findings revealed hscNPCs failed to survive long-term, but the dogs exhibited better motor function recovery. Moreover, hscNPCs remodeled the injury microenvironment shortly after transplantation by reducing inflammation and enhancing angiogenesis, leading to increased endogenous neuronal regeneration. Similarly, hUMSCs neither survive long-term nor directly reconstruct neural circuits. However, basal functional recovery and endogenous neuronal regeneration were also detected in monkeys with hUMSCs.

**Conclusions:** Exogenous short-term transplantation of stem cells in large animal SCI models does not restore basal function by directly replacing neural circuits throughout the lesion site. Rather, it does so by remodeling the lesion microenvironment in the early stages of transplantation to promote endogenous neural regeneration.

## Introduction

Spinal cord injury (SCI) is a severe disorder in the central nervous system, resulting in muscle paralysis below the level of injury, and affects over 22 million patients globally 1. Motor function impairment is generally attributed to severe damage of neurons and neural circuits caused by injury 2. Additionally, research has shown that the inhibitory microenvironment formed following injury, characterized by immune cell infiltration, blood supply destruction, cystic cavities, and scar formation, significantly hinders neural regeneration and circuit remodeling 3-8.

Cell transplantation has been a promising strategy for SCI treatment in the past decade 9,10. The common sources of graft cells include neural progenitor cells (NPCs) and mesenchymal stem cells (MSCs) 11-14. One of the main challenges in using exogenous stem cells for SCI repair is ensuring their long-term survival and integration with host cells to reconstruct neural circuits that support functional recovery. Immunodeficient mice are often used to observe and evaluate the long-term repair effects of exogenous cells at the lesion site due to their low immune rejection. However, large animal SCI models with stem cell transplantation usually administrated immunosuppressants to reduce host immune rejection and maximize the survival of exogenous cells 8,15-18. Moreover, long-term immunosuppression often leads to side effects such as infection and nephrotoxicity, making postoperative care more complicated and, in severe cases, threatening the survival of injured animals 19. Thus, addressing the immunosuppression-related issues is critical for successfully translating stem cell-based therapy into clinical SCI treatment.

Some studies have verified that animals with severe SCI showed significant behavioral improvements following stem cell transplantation, although the recovery levels are still far from satisfactory 20,21. These findings suggest that exogenous stem cells can survive, differentiate, and partially replace the lost neural circuits at the lesion site. However, a recent study on SCI repair in rhesus monkeys showed that despite administering immunosuppressants before and after transplantation, the number of exogenous cells was reduced by half within 9 months after transplantation, and the number of surviving cells continued to decline 22. In another rat SCI model, nearly 90% of the exogenous stem cells were almost undetectable within 2 months after transplantation. Without immunosuppressants, the exogenous cells would almost completely be exhausted within 2-4 weeks of transplantation 23. Interestingly, regardless of whether immunosuppressants were used, the motor function of rats showed similar improvement at 8 weeks post-transplantation. Considering that almost all exogenous cells had been exhausted by the end of the above observation period, the improvement of motor function in the cell transplant group cannot be directly attributed to the participation of exogenous cells in circuit reconstruction. Furthermore, the transplanted stem cells are likely to promote the recovery of baseline motor function in other unknown ways.

In this study, we reassessed whether recovery of baseline motor function in large animals with severe SCI directly depends on forming alternative neural circuits by exogenous cells. If exogenous stem cells cannot create long-term stable and effective bridge neural circuits, while SCI animals can still achieve recovery of baseline motor function, then long-term immunosuppression may not be necessary. Overall, hscNPCs and hUMSC were transplanted into SCI canine and monkey models without ISD. The results showed that neither hscNPCs nor hUMSCs could survive long-term in the host lesion site. In contrast, dogs and monkeys that received cell transplants exhibited baseline recovery of motor function. Although exogenous stem cells failed to reconstruct bridging neural circuits at the lesion site, the endogenous axonal and neuronal regeneration level in hscNPCs and hUMSC transplant groups was significantly higher than in control groups. In addition, hscNPCs or hUMSC transplantation could significantly promote angiogenesis and reduce scar-tissue formation. We further demonstrated that the hidden roles of stem cells early after transplantation, including immunomodulation and stimulation of neurovascular regeneration, might be primarily responsible for the aforementioned long-term neural regeneration and functional recovery. Our results also indicate that exogenous transplantation of hscNPCs or hUMSC in large animal SCI models does not restore baseline function by directly replacing neural circuits in the lesion site. Rather, it does so by remodeling the microenvironment in the early stages of transplantation to promote endogenous neural regeneration. This endogenous cell-mediated motor function recovery is not affected by immune rejection, thereby avoiding the need for prolonged immunosuppressive regimens. Uncovering this novel exogenous stem cell transplantation mechanism to promote baseline function recovery helps identify intervention targets to enhance functional recovery. It also reduces the side effects of immunosuppression, highlighting its significant potential for clinical translation.

## Results

**Figure 1:**
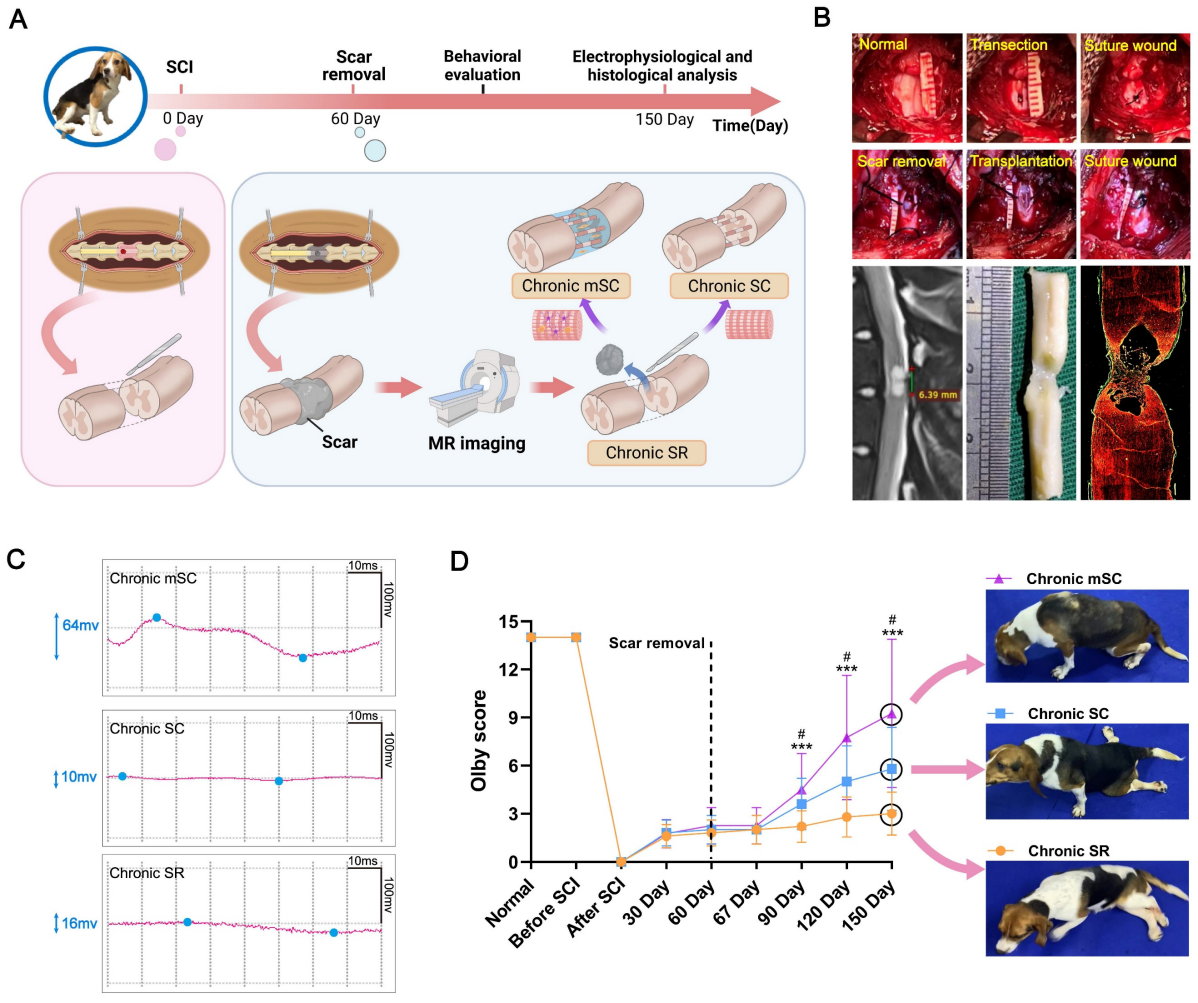
** The experiment design, surgery process of canine SCI model, neuro-electrophysiological detection, and locomotor function behavior of three groups.** (**A**) The timeline chart of spinal cord injury and analysis. (**B**) Images showing the surgery process and magnetic resonance imaging accurately define scar tissue, evidenced by immunofluorescence staining results (red: GFAP). (**C**) Motor-evoked potential (MEP) profiles of canines from different groups. (**D**) Olby scores and corresponding behavior pictures after transplantation in canines with SCI (n = 5). Two-group comparisons were analyzed using an unpaired t-test, and multiple-group comparisons were analyzed using a one-way ANOVA analysis of variance with Tukey's test. ***P (Chronic mSC vs. Chronic SR) < 0.001, ^#^*P* (Chronic mSC vs. Chronic SC) < 0.05; mean ± SEM.

In the chronic phase of SCI, large animals exhibit stable motor function scores with no significant spontaneous neural regeneration, providing a clear baseline to assess the effects of interventions 24. Thus, we set three chronic SCI canine model groups to investigate the repair effects of human fetal spinal cord neural progenitor cells (hscNPCs). Following the injury, the canines received no treatment until the second month post-injury (2 mpi), when transplantation was performed. Before transplantation, we used a well-established scar removal protocol, previously tested in humans 25, to reduce the effects of adverse microenvironment (Figure 1B). Additionally, the protocol did not impair motor function, as evidenced by unchanged behavioral scores before and after the removal 26. Several studies have also demonstrated that stem cells seeded on various scaffolds exhibit better repair effects after transplantation than cell suspension 27,28. Therefore, we pre-seeded hscNPCs on a linearly ordered collagen scaffold before transplantation (Figure 1A and Figure S1), ensuring the cells could reside at the lesion site and participate in situ. The intervention groups included hscNPCs-modified scaffolds (Chronic mSC) and scaffolds alone (Chronic SC). Furthermore, the control group (Chronic SR) underwent scar removal without further treatment. Finally, we conducted regular electrophysiological tests and behavioral assessments over five months post-injury in three groups (Figure 1C-D and Figure S2).

### Long-term repair effect of hscNPCs-scaffold transplantation

#### hscNPCs-scaffold promotes neurophysiological recovery and improves motor function

Firstly, we investigated the degree of neurophysiological recovery in three groups (Chronic SR, Chronic SC, and Chronic mSC). Three months after transplantation, stimulation of the spinal cord segments above the injury site revealed that the Chronic mSC group exhibited significantly greater potential changes compared to the two groups (Figure 1C). Additionally, we assessed the hindlimb performance of three groups using the Olby score. The animals exhibited significant hind limb paralysis post-injury, with Olby scores dropping to zero. No significant differences in motor function recovery were observed among the three groups prior to transplantation, indicating that complete SCI models of the three groups were successfully established and homogeneous (Figure 1D). Seven days after transplantation, the groups had no significant differences in kinematic performance. However, at two months post-transplantation, the Chronic mSC had higher scores than the other two groups (8 ± 4, *P* = 0.033 versus Chronic SC, *P* = 0.0007 versus Chronic SR), and this advantage persisted for three months post-transplantation (9 ± 1, *P* = 0.038 versus Chronic SC, *P* = 0.0008 versus Chronic SR; Figure 1D). Furthermore, three months after transplantation, dogs in Chronic mSC could support their weight and walk with coordinated movement of multiple joints (Movie S1). In contrast, dogs in other groups mainly exhibited movement in only one joint and dragged their hind limbs while walking (Movie S2 and Movie S3). These results showed that the hscNPCs-scaffold could lead to better hindlimb functional recovery in canines after transplantation.

#### hscNPCs-scaffold enhances functional endogenous neuronal regeneration

**Figure 2:**
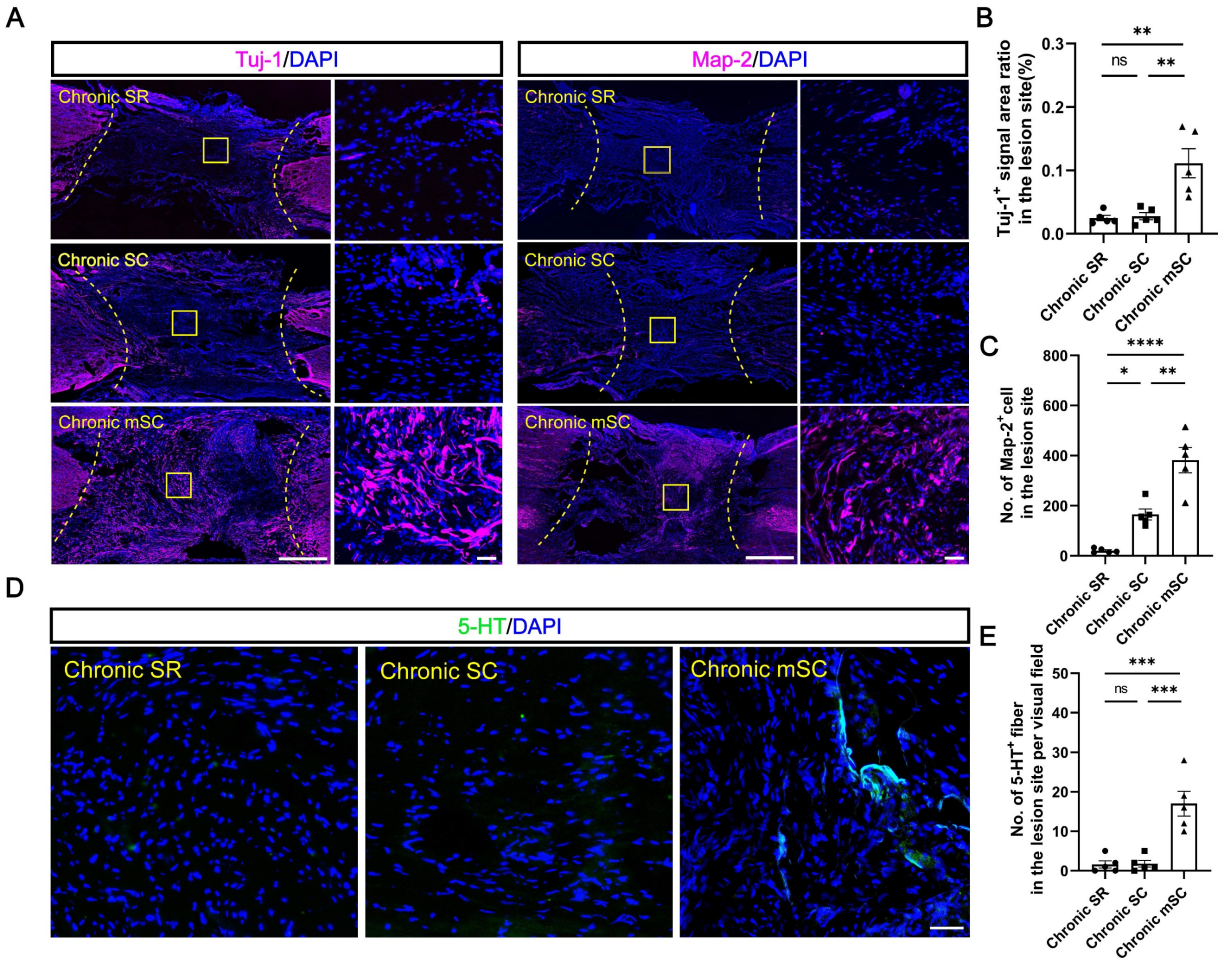
** The hscNPCs-scaffold enhances non-graft-derived endogenous neural regeneration.** (**A**) Tuj-1^+^ and Map-2^+^ neurons regenerated in the lesion site of canines in different groups at 5 mpi. Scale bar (short) = 50 μm. Scale bar (long) = 500 μm. (**B** to **C**) Quantification of Tuj-1^+^ and Map-2^+^ neurons regenerated in different groups at 5 mpi (n = 5). (**D** to **E**) The level of 5-HT^+^ neural fibers regenerated in different groups at 5 mpi (n = 5). Scale bar = 50 μm. Multiple group comparisons were analyzed using a one-way ANOVA analysis of variance with Tukey's test. *P < 0.05, **P < 0.01, ***P < 0.001, ****P < 0.0001; mean ± SEM.

To investigate the source of motor function improvement in Chronic mSC, we first examined whether hscNPCs survived at the lesion site to confirm the formation of the exogenous neural relay. Results demonstrated no MCherry^+^ graft cells surviving in the central injury area for most dogs in Chronic mSC, except for one dog with a few MCherry^+^ cells that did not co-staining with the human nuclear antigen (Figure S3A-C). Next, we compared neuronal regeneration among three groups. Chronic mSC had the most Tuj-1^+^ (0.111 ± 0.022%, Tuj-1^+^ area ratio, *P* = 0.003 versus Chronic SC, *P* = 0.002 versus Chronic SR) and Map-2^+^ (382 ± 51, Map-2^+^ cell, *P* = 0.001 versus Chronic SC, *P* < 0.0001 versus Chronic SR) neurons, with regenerated neurons nearly filling the entire injury site (Figure 2A-C). In contrast, the Chronic SR group exhibited approximately twenty Map-2^+^ neurons (21 ± 4) accumulating in the lesion area, with the Tuj-1^+^ signal area being less than one-thousandth of the lesion area (0.027 ± 0.005%), similar to Chronic SC (Figure 2A-C). However, the lesion of Chronic SC contained 166 ± 22 Map-2^+^ neurons, slightly more than the control but fewer than the cell transplantation group. We also investigated the number of 5-HT^+^ fibers, which played an essential role in the recovery of locomotor function and coordination of hindlimb movements after SCI 29. An average of seventeen serotonergic fibers (17 ± 3, *P* = 0.0004 versus Chronic SC, *P* = 0.0003 versus Chronic SR) was present in the central lesion area in Chronic mSC, in contrast to their absence in the other groups (Figure 2D-E). Overall, hscNPCs-scaffold transplantation enhanced endogenous neuronal regeneration but did not establish exogenous neural relays within three months post-transplantation.

#### hscNPCs-scaffold stimulates angiogenesis and reduces CSPG deposition in lesions

**Figure 3:**
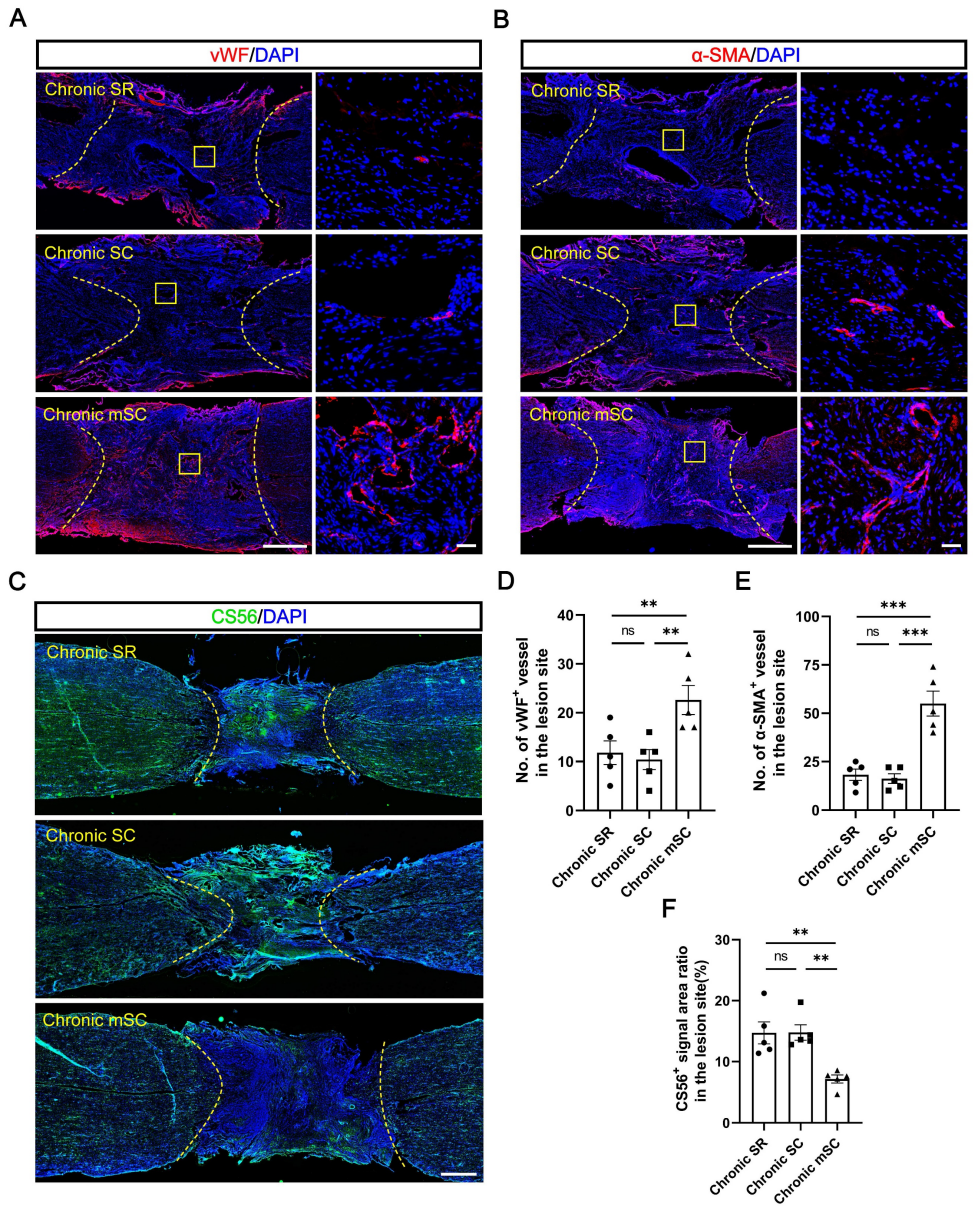
** The hscNPCs-scaffold promotes SCI repair by modulating the microenvironment.** (**A**) Regenerated vWF^+^ vessels within the lesion area were among the three groups. Scale bar (short) = 50 μm. Scale bar (long) = 500 μm. (**B**) Regenerated α-SMA^+^ vessels in the lesion site among three groups. Scale bar (short) = 50 μm. Scale bar (long) = 500 μm. (**C**) The hscNPCs-scaffold reduced chondroitin sulfate proteoglycans (CSPGs) deposition in the lesion site. Scale bar = 1000 μm. (**D** to **E**) The number of vWF^+^ (D) and α-SMA^+^ (E) vessels of the lesion site in three groups (n = 5). (**F**) Quantification of CSPGs^+^ signal area in the lesion site (n = 5). Multiple group comparisons were analyzed using a one-way ANOVA analysis of variance with Tukey's test. *P < 0.05, **P < 0.01, ***P < 0.001, ****P < 0.0001; mean ± SEM.

The results suggest endogenous neuronal regeneration in lesions may result from microenvironmental improvements. Angiogenesis and chondroitin sulfate proteoglycans (CSPGs) deposition in scar tissue have long been considered critical microenvironmental factors 30. Therefore, we evaluated the angiogenesis and CSPG deposition levels in each group. The Chronic mSC had the highest number of blood vessels, identified in the lesion center by immunostaining with 22 ± 3 vWF^+^ (vascular endothelial cell marker) (*P* = 0.012 versus Chronic SC, *P* = 0.025 versus Chronic SR) and 55 ± 6 α-SMA^+^ (vascular smooth muscle cell marker) (*P* = 0.0001 versus Chronic SC, *P* = 0.0002 versus Chronic SR) blood vessels (Figure 3A-B). In contrast, Chronic SC had fewer blood vessels in the lesion, with 10 ± 2 vWF^+^ and 16 ± 2 α-SMA^+^ blood vessels, similar to Chronic SR (Figure 3D-E).

The CSPG accumulation results contrasted, and CS56^+^ (CSPG marker) signals filled approximately fifteen percent of the lesion area in two groups (14.781 ± 1.264% for Chronic SC, 14.712 ± 1.797% for Chronic SR). In contrast, it was significantly decreased in canines transplanted with hscNPCs-scaffold, occupying less than ten percent of the area (7.167 ± 0.661%) (Figure 3C and 3F). These results indicated that the hscNPCs-scaffold could promote angiogenesis and reduce CSPG deposition in non-neural scar tissue, which benefited SCI repair.

### Short-term repair effect of hscNPCs-scaffold transplantation

At 5 mpi, we observed significantly better SCI repair in Chronic mSC compared to other groups. At this time, the hscNPCs were not detectable in the lesion. This indicated that the short-term graft intervention may be closely associated with the long-term therapeutic effects. Following SCI, animals exhibited spontaneous neuronal regeneration at the injury site, related to nestin^+^ cells at the lesion margins 31. These cells had stem cell characteristics and the capacity for neuronal differentiation and proliferation *in vitro* 32,33; they accumulated at the lesion margins during the acute phase and gradually decreased over time, disappearing entirely in the chronic phase 32. However, our study found that the first complete scar removal in the chronic phase of SCI could lead to the reappearance of nestin^+^ cells and promote the recovery of motor function. In contrast, partial scar removal did not yield these effects 26.

**Figure 4:**
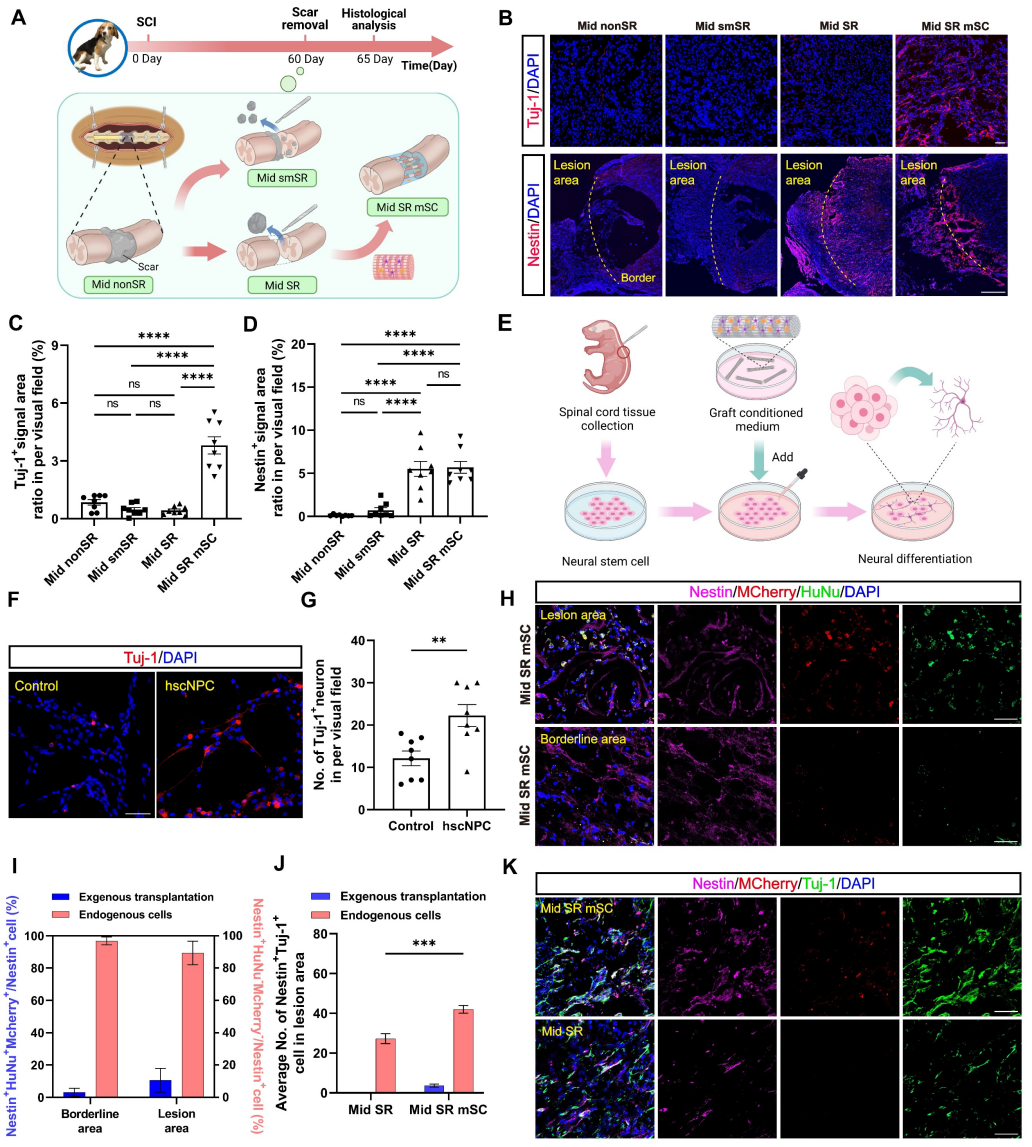
** The hscNPCs-scaffold promotes nestin^+^ cell neural differentiation *in vitro* and *in vivo*.** (**A**) The timeline chart and process of the transplantation at 2 mpi. (**B**) The nerve regeneration and endogenous cell accumulation at the edges of the lesion area in four groups. Scale bar (short) = 50 μm. Scale bar (long) = 500 μm. (**C** to **D**) Quantification of Tuj1^+^ (C) and Nestin^+^ (D) signal area in different groups' border area (n = 8 images). Eight images were randomly selected from the lesion site of three replicates in each group. (**E**) Process of experiments in vitro. (**F**) Profiles of neuronal differentiation of neural stem cells were treated with four different conditioned media. Scale bar = 50 μm. (**G**) The average number of Tuj-1^+^ neurons in two groups (n = 8 images). (**H** to **I**) Most aggregated nestin^+^ cells originated from endogenous cells in Mid SR mSC (n = 8 images). Scale bar = 50 μm. (**J**) The proportion of co-labeled cells from different sources in the lesion area of two groups (n = 8 images). (**K**) Profiles of Nestin^+^ Tuj-1^+^ co-labeled cells' accumulation in the lesion site of two groups. Multiple group comparisons were analyzed using one-way ANOVA analysis of variance with Tukey's test. Two-group comparisons were analyzed using an unpaired t-test. *P < 0.05; **P < 0.01; ***P < 0.001; ****P < 0.0001; mean ± SEM.

We then hypothesized that the long-term therapeutic effects of hscNPCs-scaffold resulted from the early interaction between hscNPCs and nestin^+^ cells. To explore this relationship, at 2 mpi, we set four groups of chronic SCI models before the transplantation (Mid nonSR, Mid smSR, Mid SR, and Mid SR mSC). The control group (Mid nonSR) received no treatment following SCI. In the Mid smSR and Mid SR groups, partial and complete scar removal was performed at 2 mpi to investigate the association between complete scar removal and nestin^+^ cells. In the Mid SR mSC group, hscNPCs-scaffolds were implanted after complete scar removal. All groups underwent histological analysis five days post-transplantation (Figure 4A).

#### hscNPCs-scaffold promotes endogenous Nestin^+^ cell neural differentiation

First, we examined whether early neural regeneration differed at the injury site following cell transplantation. Compared to three non-transplantation groups, the Mid SR mSC exhibited higher Tuj-1^+^ signals (3.813 ± 0.446%, *P* < 0.0001; Figure 4B-C), which were not co-labeled with MCherry, indicating they were not derived from the graft (Figure S4A). Similarly, immunostaining for GAP43 (growth-associated protein-43), a marker of newly generated axons, showed axonal regenerative signals occupied approximately twenty percent of the lesion area (17.991 ± 2.736%, *P* = 0.0002). In contrast, the non-transplantation group exhibited less than ten percent (2.949 ± 0.669%) (Figure S4B-C). These results indirectly supported the idea that adding graft appeared to remodel the microenvironment of the lesion site, promoting neuronal regeneration.

To confirm the effect of hscNPCs-scaffold on endogenous cells, we performed microsurgery to isolate and digest the spinal cords of P1 neonatal mice. We then extracted neural stem cells (NSCs) and cultured them *in vitro*. Meanwhile, we collected the conditioned media from pre-transplantation hscNPCs and added it to the NSCs. Culture medium mixed with scaffold served as the control. After seven days of culture, we assessed their level of neuronal differentiation (Figure 4E). The results showed that conditioned media containing hscNPCs promoted neural stem cells to differentiate more into neurons, with 22 ± 3 Tuj-1^+^ neurons per visual field (*P* = 0.007 versus control). This finding indicates that hscNPCs significantly contributed to the therapeutic effects of hscNPCs-scaffold transplantation on SCI (Figure 4F-G).

Next, we examined nestin expression at the margins of the lesion. No nestin^+^ signals were found at the margins in the partial scar removal and no treatment groups (Figure 4B). In contrast, two complete scar removal groups exhibited many nestin^+^ signals at the lesion center and margins (5.693 ± 0.691% for Mid SR mSC, 5.524 ± 0.868% for Mid SR; Figure 4B and 4D). Additionally, there was no significant difference in the number of nestin^+^ cells between the two groups (*P* = 0.996; Figure 4D). Given that some exogenous cells in the transplantation group also expressed nestin in the early period, we quantified the proportion of endogenous nestin^+^ cells and found that over 90% were not derived from the graft (93.113 ± 3.711%; Figure 4H-I). This confirmed that only complete scar removal led to the accumulation of nestin^+^ cells at the lesion, consistent with the previous study 26. Since the accumulation of nestin^+^ cells and neural regeneration occurred in the same anatomical regions, further observation revealed co-localization of nestin^+^ cells and Tuj-1^+^ neurons, with more co-stained cells in the transplantation group (46 ± 2, *P* < 0.0001 versus Mid SR; Figure 4J-K). This suggested that hscNPCs-scaffold transplantation increased endogenous neural regeneration and promoted neuronal differentiation of these nestin^+^ cells. Additionally, these regenerated neurons expressed either the excitatory transmitter marker vesicular glutamate transporter 2 (vGlut2) or the inhibitory marker glutamic acid decarboxylase 67 (GAD67) (Figure S4D-G). These findings suggested that hscNPCs-scaffold transplantation following scar removal could promote endogenous functional neuronal regeneration within five days post-transplantation.

#### hscNPCs-scaffold improves microenvironment and blood supply in lesion during the acute phase

**Figure 5:**
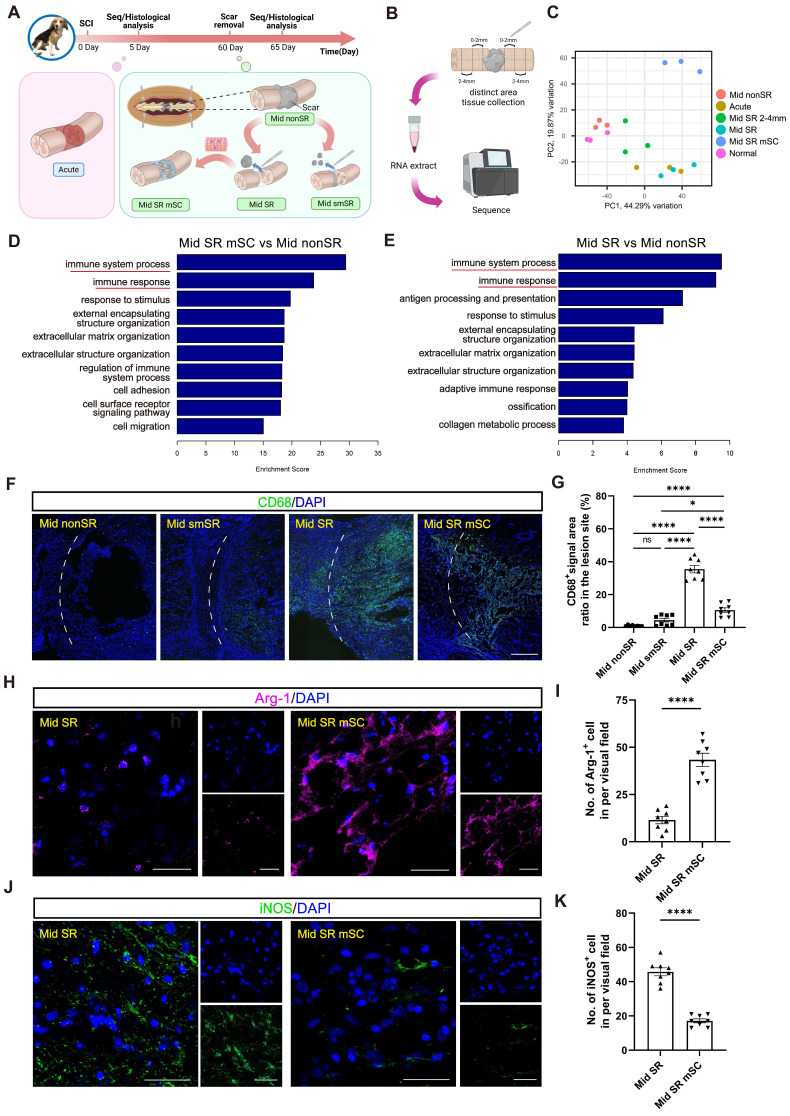
** The hscNPCs-scaffold creates a favorable immune microenvironment for regeneration.** (**A**) The timeline chart and the composition of groups in the RNA sequence at 65 dpi. (**B**) The process of RNA sequence. (**C**) PCA analysis of the six groups. Three points means three replicates in each group. (**D** to **E**) Comparison of Gene Ontology (GO) Enrichment analysis among three groups. (**F**) Profiles of inflammatory cell infiltration in the border area of four groups. Scale bar = 500 μm. (**G**) Percentage of CD68^+^ signal area in the lesion site of four groups (n = 8 images). (**H**) Profiles of Arg-1^+^ cells reside in the border area between two groups. Scale bar = 50 μm. (**I**) The number of Arg-1^+^ cells in the lesion site between two groups (n = 8 images). (**J**) Profiles of iNOS^+^ cells reside in the border area between two groups. Scale bar = 50 μm. (**K**) The number of Arg-1^+^ cells in the lesion site between two groups (n = 8 images). Multiple group comparisons were analyzed using one-way ANOVA analysis of variance with Tukey's test. Two-group comparisons were analyzed using an unpaired t-test. *P < 0.05; **P < 0.01; ***P < 0.001; ****P < 0.0001; mean ± SEM.

*In vitro* experiments and immunofluorescence staining showed that hscNPCs improved the microenvironment around endogenous cells. This promoted greater neuronal differentiation of nestin^+^ cells, which primarily accumulate at the margins. We used uninjured dogs as controls to investigate the process of transcriptomic dynamic changes in the region. We established five groups (Acute, Normal, Mid nonSR, Mid SR, Mid SR mSC) with observation points one day before and five days after each intervention. We then isolated tissues at distances of 0-2 mm from the lesion border and performed RNA sequencing (Figure 5A-B). PCA analysis showed that the transcriptomic profiles of the control group (Mid nonSR) were similar to normal spinal cord tissue but significantly different from the Acute and Mid SR clusters. We also collected tissue samples from the more distal region, specifically 2-4 mm from the border. The Mid SR 2-4 mm cluster was distributed between these two groups, demonstrating that the 0-2 mm tissue sequencing results accurately reflected the edge region's transcriptomic changes (Figure 5C). However, the cluster from the Mid SR mSC group differed entirely from the other four groups, suggesting that hscNPCs-scaffold transplantation further influenced the transcriptomic levels in the marginal tissues (Figure 5C).

**Figure 6:**
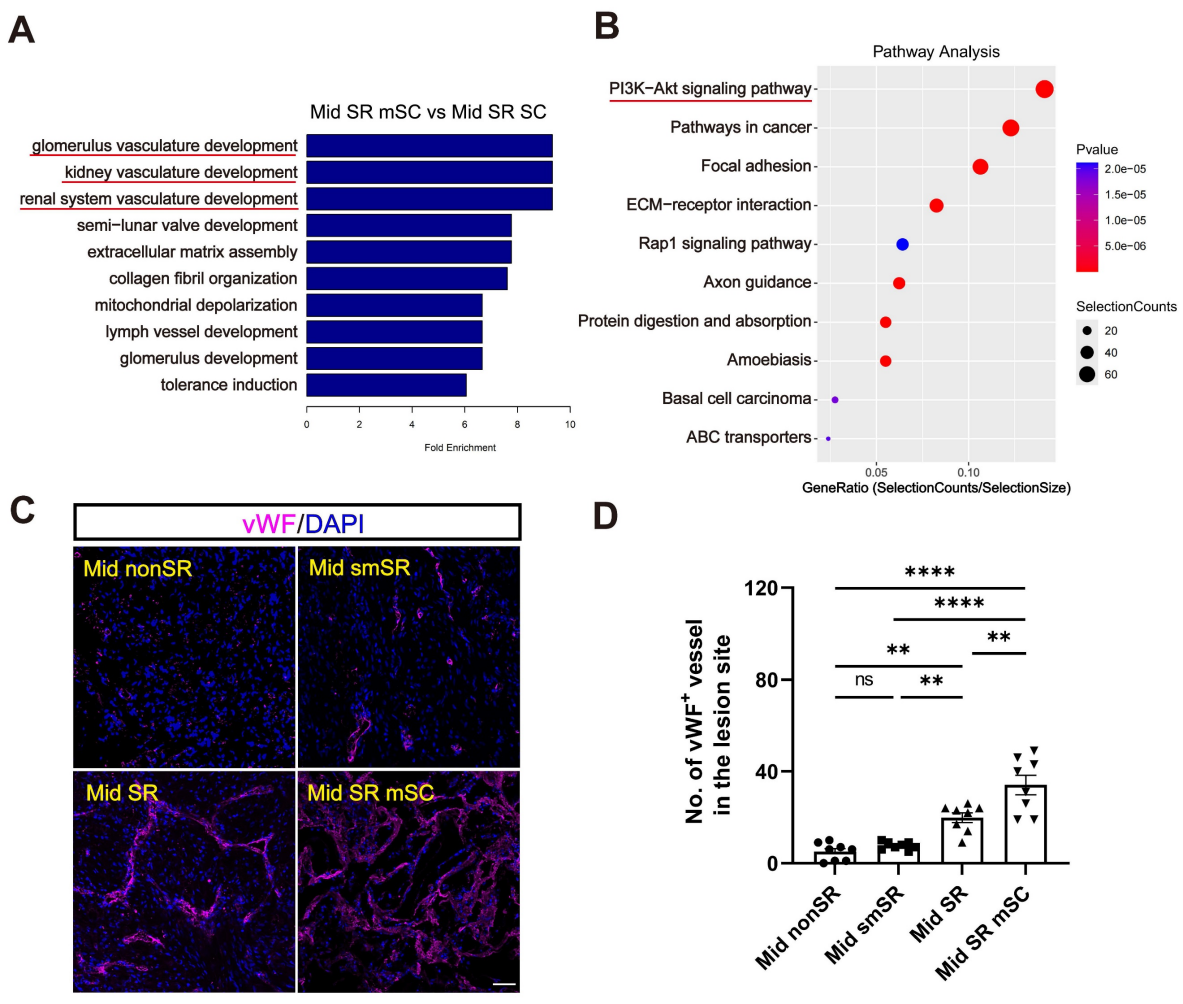
** The hscNPCs-scaffold increases angiogenesis.** (**A**) GO Enrichment analysis between the Mid SR mSC and the Mid SR group. (**B**) Pathway analysis between Mid SR mSC and the Mid SR group. (**C**) Profiles of vWF^+^ vessels were regenerated at the lesion site of four groups. Scale bar = 50 μm. (**D**) The number of vWF^+^ vessels in the lesion site of four groups. (n = 8 images). Multiple group comparisons were analyzed using a one-way ANOVA analysis of variance with Tukey's test. *P < 0.05; **P < 0.01; ***P < 0.001; ****P < 0.0001; mean ± SEM.

We performed GO enrichment analysis comparing Mid SR mSC and Mid SR with the control group (Mid nonSR). This analysis revealed a significant enrichment of differential genes related to immune responses (Figure 5D-E and Figure S5A-B). Immunostaining for CD68 (an inflammation marker) showed that complete scar removal in the chronic SCI model triggered a further inflammatory response (35.491 ± 2.223%, CD68^+^ area ratio). In contrast, hscNPCs-scaffold transplantation significantly reduced inflammation levels (10.571 ± 1.367%) (Figure 5F-G). We further identified that the grafts likely reduce inflammation by promoting the polarization of microglia or macrophages from a pro-inflammatory to an anti-inflammatory type. In hscNPC-treated canines, Arg-1^+^ signals (representing M2 type microglia and macrophages with anti-inflammatory and pro-regenerative properties) significantly increased in the lesion area and injury borders (43 ± 3, *P* < 0.0001 versus Mid SR) (Figure 5H-I). Conversely, the number of iNOS^+^ cells (representing M1 type microglia and macrophages) was lower (17 ± 1, *P* < 0.0001 versus Mid SR) (Figure 5J-K). These findings suggest that hscNPCs-scaffold play a crucial role in reducing inflammatory cell infiltration and modulating phenotypes to decrease inflammation, thereby improving the lesion microenvironment. The transcriptomic analysis also revealed significant differential expression of genes related to vascular development and angiogenesis between the Mid SR mSC and Mid SR (Figure 6A and Figure S5C). Similarly, pathway analysis showed these genes were predominantly enriched in the PI3K-AKT pathway, known to be involved in angiogenesis (Figure 6B). Furthermore, immunostaining for vWF^+^ and α-SMA^+^ vessels showed that hscNPCs-scaffold transplantation promotes early vascularization in lesions, creating a favorable environment for SCI repair (34 ± 4, *P* < 0.0001 versus Mid smSR and Mid nonSR, *P* = 0.0019 versus Mid SR; Figure 6C-D and Figure S6).

### Long-term repair effect of hUMSCs-scaffold transplantation

From the experiment in canines, we observed that hscNPCs do not repair SCI through traditional in situ differentiation of graft cells. Instead, they may promote endogenous neural regeneration and improve motor function recovery in the early phase through pro-regenerative inflammatory modulation and angiogenesis. This indicates that cell transplantation has substantial potential in remodeling the adverse microenvironment of SCI lesions and demonstrating the generalizability of these improvements is crucial for their clinical translation. Current research suggests that mesenchymal stem cells (MSCs) neurotrophic and protective effects are the main advantages of SCI repair. Although MSCs exhibit neuron-like characteristics under specific conditions, direct conversion into functional neurons has not been rigorously proven 34. Therefore, we set an experiment to transplant human umbilical cord-derived MSCs (hUMSCs) into a large animal acute SCI model. Without considering differentiation into neurons for neural circuit reconstruction, we aimed to analyze whether hUMSCs, like NSCs, can remodel the microenvironment during the early stages post-transplantation. In this experiment, we used rhesus monkeys, anatomically closer to humans. We evaluated the effects of hUMSC transplantation on endogenous neural regeneration and functional improvement using an acute SCI model that benefits repair.

**Figure 7:**
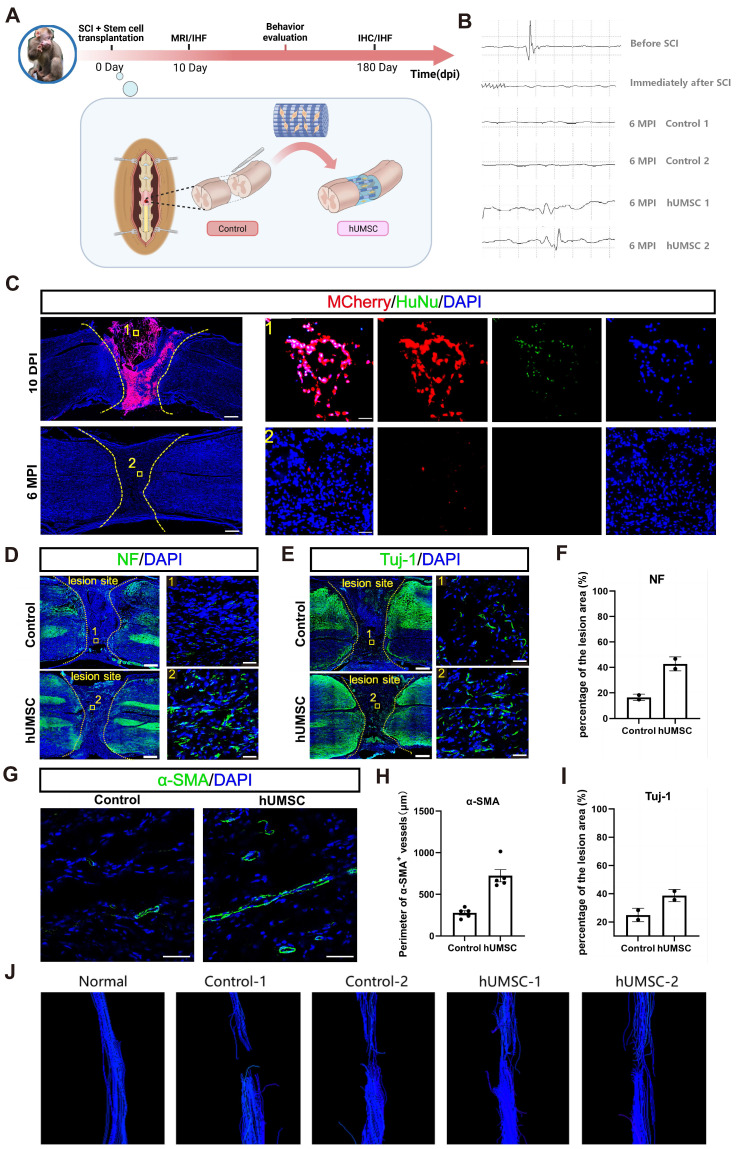
** The hUMSCs-scaffold improves angiogenesis and endogenous neural regeneration at 6 mpi.** (**A**) The timeline chart of spinal cord injury and the composition of monkey groups. (**B**) The profiles of motor evoked potentials (MEP) in monkeys before and after injury between two groups. Each horizontal grid represents 5ms, and each vertical grid represents 100 µV. (**C**) Survival of grafted cells in the lesion site of monkeys at 10 dpi and 6 mpi. Scale bar (left)= 500 μm. Scale bar (right) = 50 μm. (**D** to **E**) NF^+^ (D) and Tuj-1^+^ (E) axons regenerated in the lesion site of monkeys in different groups at 6 mpi. Scale bar (left) = 500 μm. Scale bar (right) = 50 μm. (**F**) Quantification of NF^+^ axons of monkey regenerated in different groups at 6 mpi (n = 2). (**G**) α-SMA^+^ vessels regenerated in the lesion site of monkeys between two groups at 6 mpi. Scale bar = 50 μm. (**H**) Quantification of α-SMA^+^ vessels of monkeys between two groups at 6 mpi (n = 5 images). Eight images were randomly selected from the lesion site of two monkeys in each group. (**I**) Quantification of Tuj1^+^ axons of monkey regenerated in different groups at 6 mpi (n = 2). (**J**) Diffusion tensor imaging (DTI) assessments of monkeys before and after injury between two groups. Error bars represent the standard error. Two-group comparisons were analyzed using unpaired t-test; mean ± SEM.

Eight adult monkeys were randomly divided into two groups after spinal cord complete transection at T10. Monkeys in the control group were left untreated, and the dura mater and muscle skin were sutured. During the six-month observation following surgery, magnetic resonance imaging (MRI), electrophysiological tests, and behavioral assessments were performed regularly in the rhesus monkeys (Figure 7A). In the cell transplantation group, collagen scaffolds seeded with 1×10^7^ hUMSCs were immediately transplanted into the lesion site post-injury (Figure S7). hUMSCs were isolated and expanded as described in our previously published work 35. To observe the survival and residence after transplantation, hUMSCs were labeled explicitly with MCherry^+^ red fluorescent protein before seeding into the scaffold (Figure S7A).

#### hUMSCs-scaffold benefits neurophysiological recovery

Similar to the former protocol in canines, we examined whether hUMSC transplantation could improve neurophysiological recovery and further benefit motor function recovery. Motor evoked potentials (MEPs) were usually detected to assess the integrity of motor pathways 35. Preoperative stimulation of the T9 segment detected well-defined MEPs in the muscles of the lower limbs, which vanished immediately after complete spinal cord T10 transection, indicating a successful establishment of a complete SCI model (Figure 7B). Subsequently, we assessed the recovery of MEPs in monkeys from both control and hUMSCs-scaffold transplantation groups at 6 mpi. Notably, effective MEPs were absent in the control group. In contrast, the transplantation group exhibited restored MEPs at approximately one-third of their preoperative levels (Figure 7B). The above results demonstrate that the hUMSCs-scaffold promotes neurophysiological recovery. However, in terms of behavioral performance, the monkeys that received the transplants did not exhibit the same degree of stepping ability recovery as the canines. This may indicate that there is still a discrepancy in the short-term survival of hscNPCs and hUMSCs with regard to their efficacy in SCI repair.

#### hUMSCs-scaffold facilitates functional axon regeneration

Motor function recovery of muscle recruitment and locomotion was correlated with the anatomical recovery of the neural network 36. We then analyzed the increased degree of neural regeneration in the lesion site and inspected the survival of hUMSCs in the transplantation group. Like the results with hscNPCs in canines, MCherry^+^ graft cells densely populated the injury site at 10 dpi but disappeared at 6 mpi (Figure 7C). This suggests that hUMSCs survived only in the short term and that any subsequent observed neural regeneration was not directly attributed to the graft cells.

We then compared the regeneration profiles of axons and neural fibers in lesion sites within the control and transplantation groups at 6 mpi. It was revealed that there are few NF^+^ (neurofilament markers) axons and Tuj1^+^ (new-born neuronal marker) neurons detected in the control group (Figure 7D-E). In contrast, the hUMSCs-scaffold group exhibited NF^+^ and Tuj1^+^ signals twice as strong as those in control, with regenerated axons and neurons nearly covering the entire lesion site (Figure 7D-E). Moreover, we conducted diffusion tensor imaging (DTI) assessments to validate the reliability of the above immunostaining results. In the hUMSCs group, significantly more fibers bridged the ends of the injury site. In comparison, the control group had relatively few such connections, demonstrating that the hUMSCs scaffold facilitates axonal regeneration (Figure 7J).

#### hUMSCs-scaffold improves the blood supply and reduces scar formation in the lesion site

Previous research showed that MSC transplantation can promote the remodeling and regeneration of blood vessels following SCI 37-39. Accordingly, we examined the improved degree of angiogenesis by performing an immunostaining analysis of α-smooth muscle actin (α-SMA) and vWF in the spinal cord tissues of monkeys in the control and transplantation group at 6 mpi. Results showed that the average α-SMA^+^ vessel perimeter in the transplantation group was nearly three times larger than in the control group. However, the number of vWF^+^ vessels did not differ significantly between two groups (Figure 7G-H and Figure S8).

Neural scar tissue formed in the lesion center after spinal cord injury secretes chondroitin sulfate proteoglycan (CSPG) to inhibit axonal regeneration and functional recovery. Additionally, previous studies have demonstrated that MSC transplantation can inhibit excessive glial scar formation and further promote axon regeneration by secreting neurite factor 40-42. Thus, we detected the deposition of extracellular matrix components such as CSPG in the injured area of monkeys at 6 mpi. Notably, the CSPG-positive signal filled almost the entire lesion area in control monkeys, while it was scarcely detected in monkeys transplanted with hUMSCs-scaffolds (Figure S9A). These findings indicated that hUMSC transplantation significantly reduced CSPG expression at the lesion site.

### Short-term repair effect of hUMSCs-scaffold transplantation

At 6 mpi, we observed that the SCI repair effects did not originate directly from MSCs. One study reported that MSCs were more likely to support and protect endogenous cells than differentiate into fully functional neural cells 36. Based on the short-term observation of tremendous surviving graft cells (Figure 7C), we hypothesized that the long-term repair effects, similar to our findings in dogs, may result from the short-term regulatory influence of MSCs on endogenous cells.

#### hUMSCs-scaffold stimulates axon regeneration within a short period

**Figure 8:**
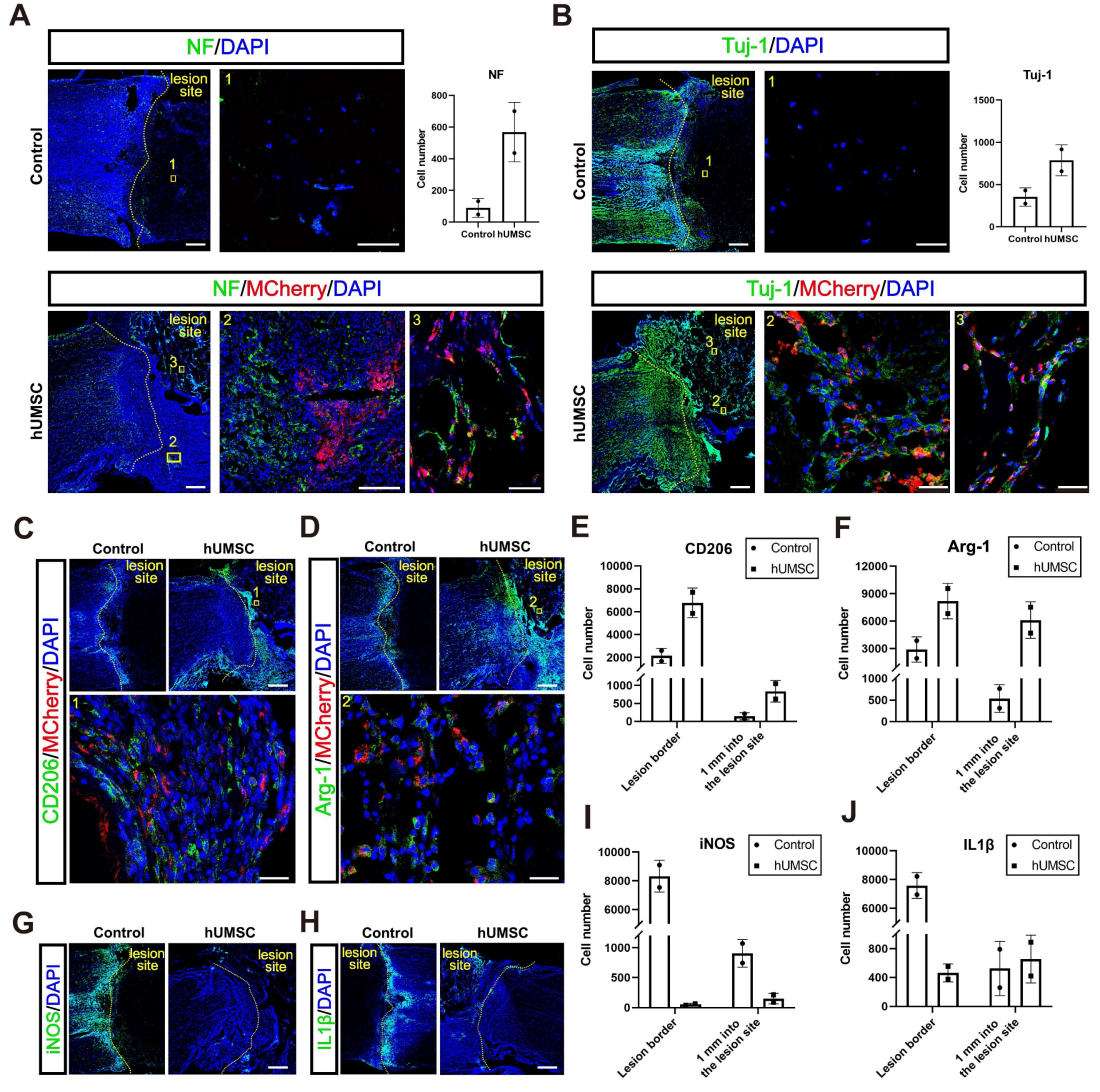
** The hUMSCs-scaffold promotes axon regeneration and alleviates the inflammatory response within 10 dpi.** (**A** to **B**) NF^+^ (A) and Tuj1^+^ (B) axons regenerated in the lesion site of monkeys in different groups at 10 dpi. Scale bar (left) = 500 μm. Scale bar (right) = 50 μm. (**C** to **D**) CD206 (C) and Arg-1 (D) positively stained cells in the lesion site of monkeys in each group at 10 dpi. Scale bars indicate 500 and 50 μm, respectively. (**E** to **F**) Quantification of CD206 (E) and Arg-1 (F) positively stained cells in the lesion site of monkeys in each group at 10 dpi. (**G** to **H**) iNOS (G) and IL-1β (H) positively stained cells in the lesion site of monkeys in each group at 10 dpi. Scale bar = 500 μm. (**I** to **J**) Quantification of iNOS (I) and IL-1β (J) positively stained cells in the lesion site of monkeys in each group at 10 dpi; mean ± SEM.

Firstly, we conducted Tuj-1 and NF immunostaining analysis in the control and hUMSCs-scaffolds transplantation groups at 10 dpi. Monkeys in the transplantation group showed more Tuj-1^+^ and NF^+^ axons and nerve fibers in the lesion sites (Figure 8A-B). Moreover, the regenerated Tuj-1^+^ and NF^+^ nerve fibers in the lesion center were more prominently visible where MCherry^+^ hUMSCs were present, but co-labeling was not detected (Figure 8A-B). This suggests that the extension of regenerated nerve fibers exhibits a cell-based tendency. From the same observation shown in the immunostaining result of GAP43, there was no difference with GAP43^+^ axons at the lesion border. However, around 500 GAP43^+^ axons were in the lesion site of the hUMSCs group, and the control had only about 400 (Figure S9B). We further analyzed the relationship between endogenous neural regeneration and nestin^+^ cells. Like hscNPCs, hUMSCs promoted more neuronal differentiation of nestin^+^ cells, as evidenced by the Tuj-1^+^ neurons co-labeled with nestin in the hUMSC-transplanted group compared to the control (Figure S10).

#### hUMSCs-scaffold alleviates the inflammatory response in the acute phase after injury

We speculated whether hUMSCs, similar to hscNPCs, could create a conducive environment for regeneration by modulating the inflammatory response and promoting the polarization of microglia and macrophages from a pro-inflammatory (M1) phenotype to an anti-inflammatory (M2) phenotype. Our results showed that the number of cells expressing CD206 and Arg1 (representing M2-type microglia and macrophages with anti-inflammatory and pro-regenerative properties) was significantly higher in the lesion area and injury borders of the hUMSCs-treated monkeys (Figure 8C-F). Conversely, the presence of cells expressing iNOS and IL-1β (representing M1-type microglia and macrophages with pro-inflammatory and anti-regenerative properties) was notably higher in the lesion area and injury borders of control monkeys (Figure 8G-J). Therefore, these results indicated that the transplantation of hUMSCs may provide a basis for regeneration at the injury site by modulating the types of inflammatory cells.

## Discussion

Cell transplantation is considered a promising strategy for addressing spinal cord injury (SCI), including stem cells, supportive cells, and immune cells 10. Importantly, these approaches could enhance host motor function by replacing damaged neural cells, secreting neurotrophic factors, modulating gliosis and scar formation, preventing cavity formation, and promoting axonal regeneration 43,44. However, avoiding graft cell death due to immune rejection remains a significant challenge post-transplantation 45,46. Immunosuppressive drugs (ISD) are commonly used to address this issue, including calcineurin inhibitors (cyclosporine A, tacrolimus), antimetabolites (mycophenolate, azathioprine), and glucocorticoids. A recent study on human NSC transplantation into cervical SCI in non-human primates (rhesus monkeys) demonstrated long-distance axon regeneration from graft cells, synapse formation with host neurons, support for host corticospinal axon regeneration, and recovery of forelimb motor function 22. Despite using a triple-therapy immunosuppressive regimen (MMF, tacrolimus, prednisone) to maintain long-term graft cell survival for nine months, the study observed a continuous decline in graft cell density and total cell number. This suggests chronic immune rejection cannot be entirely avoided post-transplantation, even with ISD.

Additionally, complete cell loss and decreased motor function remain possible in the years following transplantation. Another study focusing on the impact of ISD on long-term graft survival found that ISD promoted early survival and inhibited apoptosis of transplanted hscNPCs, maintaining the early differentiation at the lesion and better locomotor performance. However, there was a significant reduction in graft-derived neurons and new axons by two months, with no notable difference in motor scores between the ISD and non-ISD groups. This suggests that ISD-protected hscNPCs may contribute to early functional recovery. Still, a chronic SCI-negative microenvironment or chronic immune rejection effects limit the long-term protective efficacy of ISD. Furthermore, even with the use of immunosuppressive drugs to preserve graft cells, the animals' motor recovery remains at a low level. Similar motor performance was observed without these drugs following transplantation. Considering that long-term ISD treatment can lead to side effects such as infections, decreased host survival rates, and malignancies, the benefits of ISDs are questionable 47. Notably, researchers must carefully consider the application of immunosuppressants. Meanwhile, to determine the long-term protective effects of ISD, future research should use appropriate SCI models, extend observation periods to several years, and record motor performance. ISD's long-term ideal protective effect should result in stable cell density and total cell numbers at multiple time points (1, 2, and 4 years) without accompanying declines in host motor performance.

The improvement in motor function from cell transplantation in SCI models can be attributed to endogenous and exogenous factors and is associated with resident neural cells in the lesion area 13. Endogenous neural regeneration, which can ignore the side effects of immune rejection, holds greater potential than exogenous recovery. Studies have shown that nestin^+^ cells accumulate at the lesion margins during the acute phase post-SCI, exhibiting stem cell-like capabilities for differentiation and proliferation in vitro, which gradually diminish over time 32,33. This accumulation can reoccur even during the chronic phase of SCI by completely removing the scar tissue 26. Regulating these cells' proliferation and neuronal differentiation in vivo presents new opportunities for SCI repair. However, further experiments revealed that subsequent scar removal after the initial chronic phase SCI did not stimulate the re-accumulation of nestin^+^ cells, indicating that these resident stem cells require external intervention due to the adverse microenvironment or other factors. Our research also showed that although exogenously transplanted hscNPCs died within five months, they promoted the differentiation of nestin^+^ cells generated by scar removal in the chronic SCI model. As such, it was able to enhance endogenous motor function recovery. Although the underlying mechanism of this promotion is unclear, it deserves further investigation.

Regarding the effect of exogenous cells on endogenous function recovery, we noticed that, in a previous study, researchers modified the surface of transplanted cells with the diphtheria toxin (DT) receptor and selectively eliminated them two weeks before the end of the observation period to determine if behavioral improvements were due to exogenous graft cells 48. After elimination, the mice's hind limb motor function remained at a certain level, similar to the non-transplanted group, suggesting that the remaining motor function likely stemmed from endogenous neural regeneration. However, we found that using mouse forebrain NSCs in this study had a limited effect on improving endogenous motor function, differing significantly from our findings. From our research, three months post-transplantation in a chronic SCI model, beagles showed significantly higher Olby scores than the control group, indicating enhanced endogenous recovery not derived from the graft. Similar results were observed in another study, where adding hscNPCs to a collagen scaffold and ISD further increased motor function scores 23. While different SCI animal models can lead to analytical variations, these findings suggest that graft cells from various sources may have varying trophic effects on surrounding host cells.

## Methods

### Ethic statement

All animal were provided and accommodated at the experimental animal center in Hunan Puruima Medicine Research Center (Hunan, China). And experiments were completed under the guide that the Care and Use of Laboratory Animals from the National Institutes of Health (NIH) and were approved by the Animal Care and Use Committee of Hunan Puruima Medicine Research Center (Hunan, China). All animal procedures were in accordance with the NIH's Guide for the Care and Use of Laboratory Animals. The human cells used were derived from aborted embryonic tissue, which are from legally terminated pregnancies. The study was reviewed and approved by the Ethics Committee of Hunan Puruima Medicine Research Center (Approved number: IACUC-2019027026, IACUC-2022(5)018).

### hscNPCs-scaffold and hUMSCs-scaffold construction

To construct the hscNPCs-scaffold, hscNPCs or hscASs were dissociated and seeded on the collagen scaffold, 1.6 × 10^6^ hscNPCs was firstly seeded on the collagen scaffold, and then 4 × 10^5^ hscAS were seeded to promote hscNPCs adhesion, survival, and outgrowth. Both cells were labeled with MCherry^+^ red fluorescent protein before being seeded onto the scaffold. The appropriate hscNPCs proliferation medium was added until the material was covered, and the material and cells were incubated for 2 h. After that, another 2 ml of proliferation medium was added 30. Finally, cell proliferation medium was added for further coculture. After 7 days, the hscNPCs-scaffold were transferred into the differentiation medium for in vitro differentiation analysis, or they were transplanted to repair SCI in dogs.

For hUMSCs-scaffold construction, hUMSCs were labeled with MCherry^+^ red fluorescent protein before being seeded onto the scaffold. The umbilical cord tissue was washed in a Petri dish containing 40 mL sodium chloride with sterile forceps to remove blood. After the blood vessels and amnion were removed, Wharton's jelly was collected in a new Petri dish. The Wharton's jelly was then sliced into small pieces with 1 mm in diameter before transferred to culture flasks which were covered with glass slides to prevent from floating. After adhered to the flasks, the hUMSCs were cultured in serum-free MesenCult-XF medium and FBS bases DMEM complete medium (Stemcell, Vancouver, Canada) at 37 °C in a humidified atmosphere with 5% CO2. Additionally, the medium was changed every 3-4 days. hUMSCs were digested with 0.05% trypsin/EDTA before passage. The morphology of hUMSCs were observed and recorded under a microscope 49.

### The culture and isolation of hscNPCs and hUMSCs

According to the Code of Ethics of the World Medical Association (Declaration of Helsinki), Our source of human embryonic tissue and umbilical cord tissue were from legally terminated pregnancies.

In this study, hscNPCs were obtained from human fetal spinal cord tissue using a previously described method 49. Briefly, the excised intact spinal cord tissue was digested with protein hydrolase and collagen hydrolase (A6964, Sigma-Aldrich). The digestion was stopped with an appropriate amount of DMEM/F12 medium. The cells were resuspended by centrifugation and then cultured in cell culture dishes with proliferation medium suitable for hscNPCs. The hscAS were obtained from selectively aborted human fetuses. The intact spinal cord tissue was carefully dissected from the fetus. After complete removal of meningeal and vascular tissue, the tissue was dissociated into a single cell suspension and centrifuged at 500g for 5 min. All cells were resuspended in hscAS proliferation culture medium, and incubated at 37℃ in 5% CO2. After two rounds of purification, hscASs were passaged and used for transplantation.

The umbilical cord tissue from human was initially cleansed in a Petri dish with several sterile sodium chloride to eliminate blood. Wharton's jelly was gathered in a fresh Petri dish with the removal of blood vessels and amnion, and then sliced it into 1 mm diameter. Subsequently, transferred it to new culture flasks which were shielded by glass slides to prevent floating. After adhered to the flasks, hUMSCs were cultured using serum-free MesenCult-XF medium and FBS-based DMEM complete medium (Stemcell, Vancouver, Canada) at 37 °C with 5% CO2, and renewed the medium every 3 days. 0.05% trypsin/EDTA were used to digest before passage.

### Surgery procedures, transplantation, postoperative care, and rehabilitation

Adult male beagles, aged 2-3 years and weighing 8-9 kg, were used in our study. The beagles were maintained at 22-25°C and 40-70% humidity. Prior to surgery, the beagles had to be kept in an animal room for at least 3 weeks. After anaesthesia, the beagles were extubated and supported by mechanical ventilation. Anaesthesia was maintained with fentanyl (7-10μg/kg/h intravenously) and propofol (2.5mg/kg intravenously). Intravenous saline was also used to maintain blood volume. Respiratory rate, heart rate and oxygen saturation were monitored during the surgery. Under aseptic conditions, a 4-6 cm longitudinal incision was made at T8-T10. The erector spinae muscle was dissected away from the spinous process. A 1-2 mm section of the spinal cord was resected at T8-T9. After hemostasis with a gelatin sponge, the dura mater was closed with 4-0 surgical sutures and the muscle tissue and skin surrounding the spinal cord were closed in layers. Three months after surgery, spinal cord injured dogs were randomly divided into three groups for secondary surgery, which was performed as follows: surgical sutures were cut at the original position, the dura mater was opened, and 4-6 mm of scar tissue was excised through the scar area shown by magnetic resonance results. The dura mater, paravertebral muscles and skin were then closed in layers with sutures. As complete spinal cord transection results in loss of bladder function, we employed professionals to squeeze the beagle's bladder 2-3 times a day to assist with urination.

The detailed surgery procedure for rhesus monkeys was described as previously reported 18. Briefly, anesthetized the monkeys and provided standard surgery conditions according to prior protocol, and a 5-6 mm long segment of the spinal cord in T10 was excised using previously reported methods 50. In the cell transplantation group, collagen scaffolds seeded with 1 × 10^7^ hUMSCs were immediately transplanted into the lesion site post-injury, with no treatment in control group. Finally, dura, paraspinal muscles, and skin were closed in layers with sutures. Given that a complete transection could lead to paraplegia and loss of bowel and bladder function, we enlisted specialized staff to ensure the emptying of the monkeys' bladders 2-3 times a day.

### Spinal cord Motor evoked potential (MEP) detection

Spinal cord motor evoked potentials (MEPs) were detected in Beagles and rhesus monkeys as previously described 18,26. The anaesthetic protocol was the same for surgery and electrophysiological testing. To determine evoked motor potentials, stimulating electrodes were placed on the scalp and recording electrodes were placed on target muscles, including the rectus abdominis, and then stimulated with a single pulse to produce a neuroelectric signal. The location of the response to evoked motor potentials was recorded.

## Supplementary Material

Supplementary materials and methods, figures.

Movie S1.

Movie S2.

Movie S3.

## Abbreviations

α-SMA: α-smooth muscle actin
CSPGs: chondroitin sulfate proteoglycans
DT: diphtheria toxin
DTI: diffusion tensor imaging
GAD67: glutamic acid decarboxylase 67
hscNPCs: human spinal cord neural progenitor cells
hUMSCs: human umbilical cord mesenchymal stem cells
hscAS: human spinal cord astrocyte
ISD: immunosuppressive drugs
MRI: magnetic resonance imaging
MSCs: mesenchymal stem cells
MMF: mycophenolate mofetil
NPCs: neural progenitor cells
SCI: spinal cord injury
SCMEP: spinal cord motor evoked potentials
vGlut2: vesicular glutamate transporter 2
