# Supplementary material for: Revisiting the unobtrusive role of exogenous stem cells beyond neural circuits replacement in spinal cord injury repair
Source: Theranostics. 2025 Jan 2;15(4):1552–69. doi: 10.7150/thno.103033 (PMC11729559; doi:10.7150/thno.103033)
Supplement: Supplementary file 1 — Supplementary materials and methods, figures. [file thnov15p1552s1.pdf]

Supporting Information

Revisiting the unobtrusive role of exogenous stem cells beyond neural circuits replacement in spinal cord injury repair

Authors:

Runlin Wen[1,2†], Ge Long[3†], Xinghui He[2†], Kai Zhang[1,2], Wanrong Ma[1], Yeyu Shen[1], Zhifeng Xiao[4], Yannan Zhao[4], Dingyang Liu[2*], Jianwu Dai[4*], Xing Li[1*].

The Supporting Information includes Materials and Methods, Fig S1 to S10 for multiple supplementary figures, Movies S1 to S3 and Supplementary References.

Materials and Methods

Preparation of collagen scaffold

The collagen scaffold was prepared as previously described [26]. At first, the dehairing hides from ox were immersed in a 0.5 M acetic acid solution at 4°C. Then, the samples were mixed in a blender for 15 min and neutralized with 4 M NaOH to obtain a homogeneous collagen solution. After dialysis and freeze-drying, collagen scaffolds were obtained. To produce a linear porous structure, the collagen scaffolds were cut into pieces and permeated with 40 mL of MES (pH 6.5) solution containing 0.6 mg/mL of N-hydroxysuccinimide and 1 mg/mL of 1-ethyl-3-(3-dimethylaminopropyl) carbodiimide, and the particles were washed. For scanning electron microscope (SEM) observation, the collagen scaffolds or cell-loaded collagen scaffolds were washed three times with PBS and then fixed in 2.5% glutaraldehyde for about 90 min, followed by ethanol gradient dehydration and freeze-dried for 2 days. Finally, the dried samples were coated with gold and examined under SEM and X-ray micrograph (Zeiss, Xradia 410 Versa) exhibiting the surface and internal structures of a single

fiber, respectively (Figure S1D).

Collagen hydrogel for monkeys were prepared for cell transplantation. In brief, freshly harvested bovine aponeurosis was incubated with 1% tri(n-butyl) phosphate for 48 h. The resulting collagen was then immersed in 1% trypsin for 1 h to remove cells and other proteins. After being washed in deionized water, the collagen was dissolved in 0.5 M acetic acid for 24 h and then dialyzed in deionized water for 10 days, lyophilized and dissolved in normal saline to give a 30 mg/ml collagen hydrogel for hUMSCs loading. Collagen scaffold with hUMSCs were lyophilized, sputter-coated with gold and observed by scanning electron microscopy (SEM).

Immunostaining

The previous study had performed immunostaining method [49]. Collagen scaffolds seeded with hscNPCs and hscASs were fixed with 4% paraformaldehyde for 15 minutes at room temperature, washed three times with phosphate buffer saline (PBS), and blocked with 5% donkey serum containing 0.2% Triton X-100 for 1 hour at room temperature. The primary antibody was diluted with the blocking solution and incubated overnight, followed by incubation with the appropriate secondary antibody for 1 hour at room temperature.

Behavioral assessment

The study used the Olby score to assess functional motor recovery after spinal cord injury in dogs, and detailed scoring rules of the Olby score system were described as previously reported [51]. Two individuals blind to the experimental conditions recorded one Olby score for each dog at three groups after surgery. During the whole assessment, the canines moved freely in an open field and were rated on the basis of their ability for spontaneous or voluntary Hindlimb motion.

Statistical analyses

Data are presented as mean ± SEM. GraphPad Prism 9.5.1 (733) was used for all statistical analyses. When more than two sets of data were compared, one-way ANOVA analysis was performed, and Tukey's test was used for post hoc analysis to correct for multiple comparisons. Two-group comparisons were analyzed using unpaired t-test. p-values < 0.05 were considered significant.

Magnetic Resonance Imaging (MRI) and Diffusion Tensor Imaging (DTI)

Magnetic resonance imaging (MRI) and diffusion tensor magnetic resonance imaging (DTI) of all animals were accomplished by a 3.0 T MRI scanner (Siemens, Germany). MRI and DTI were used to assess the regeneration of spinal cord nerve tracts at 6 months after surgery.

RNA-Seq and Bioinformatics Analysis

After anesthesia, 2 mm spinal cord segments were taken from each canine, sourced from areas 0-2 mm and 2-4 mm away from the lesion border, and total RNA was extracted. RNA high throughput sequencing was performed by Cloud-Seq Biotech (Shanghai, China). Briefly, total RNA was used for removing the rRNAs with GenSeq rRNA Removal Kit (GenSeq, Inc.). Then, the rRNA-depleted samples were subjected to library construction with GenSeq Low Input RNA Library Prep Kit (GenSeq, Inc.) according to the manufacturer's instructions. Libraries were controlled for quality and quantified using the BioAnalyzer 2100 system (Agilent Technologies, Inc., USA). Library sequencing was performed on an Illumina Novaseq instrument with 150 bp paired end reads. Paired-end reads were harvested from Illumina NovaSeq 6000 sequencer and were quality controlled by Q30. After 3′ adaptor-trimming and low-quality reads removing by cutadapt software (v1.9.3), the high-quality clean reads were aligned to the reference genome with hisat2 software (v2.0.4). Then, HTSeq

software (v0.9.1) was used to get the raw count, and edgeR was used to perform normalization; then, differentially expressed mRNAs were identified by p-value and fold change. GO and Pathway enrichment analysis were performed based on the differentially expressed mRNAs.

Culture and Isolation of NSCs

The telencephalons or the spinal cord were dissected from newborn mouses. After striping the meninges, tissues were cut into 1 mm^3 pieces, and digested in 0.25% trypsin at 37 °C for 40 min. Then trypsin inhibitor was added to stop the digestion, followed by centrifugation at 500g for 5 min. The cell suspension was cultured in 25 cm^2 tissue culture flask (Corning, USA) in serum-free DMEM/F12 medium containing 20 ng/mL bFGF (Peprotech Asia, Rehovot, Israel), 20 ng/mL EGF (Peprotech Asia), 2% B27 (Invitrogen, GIBCO, NY, USA), 30% glucose (Sigma, USA), and 1.83 mg/mL heparin (Sigma, USA). After 7-day culture, neurospheres were enzymatically dissociated to single cells for the following experiments. The NSCs were resuspended in adhesion medium containing 10% FBS and 3×10^5 cells were seeded on 96-well plate pre-coated with 100 g/ml poly-l-lysine. After one day culture, the adhesion medium was removed. After washing three times with PBS, conditioned media was added. Then cells were fixed after 6 days, followed by immunofluorescence staining and subsequent analysis.

Transfection of hscNPCs and hUMSCs with lentivirus

The hscNPCs and hUMSCs were transfected with lentivirus carrying red fluorescent protein gene (MCherry). Briefly, at 40-50% confluence, the supernatant was replaced with 7 ml of fresh culture medium containing lentivirus. Then, 10 h later, 8 ml of culture medium was added and incubated for another 72 h before passaging [30].

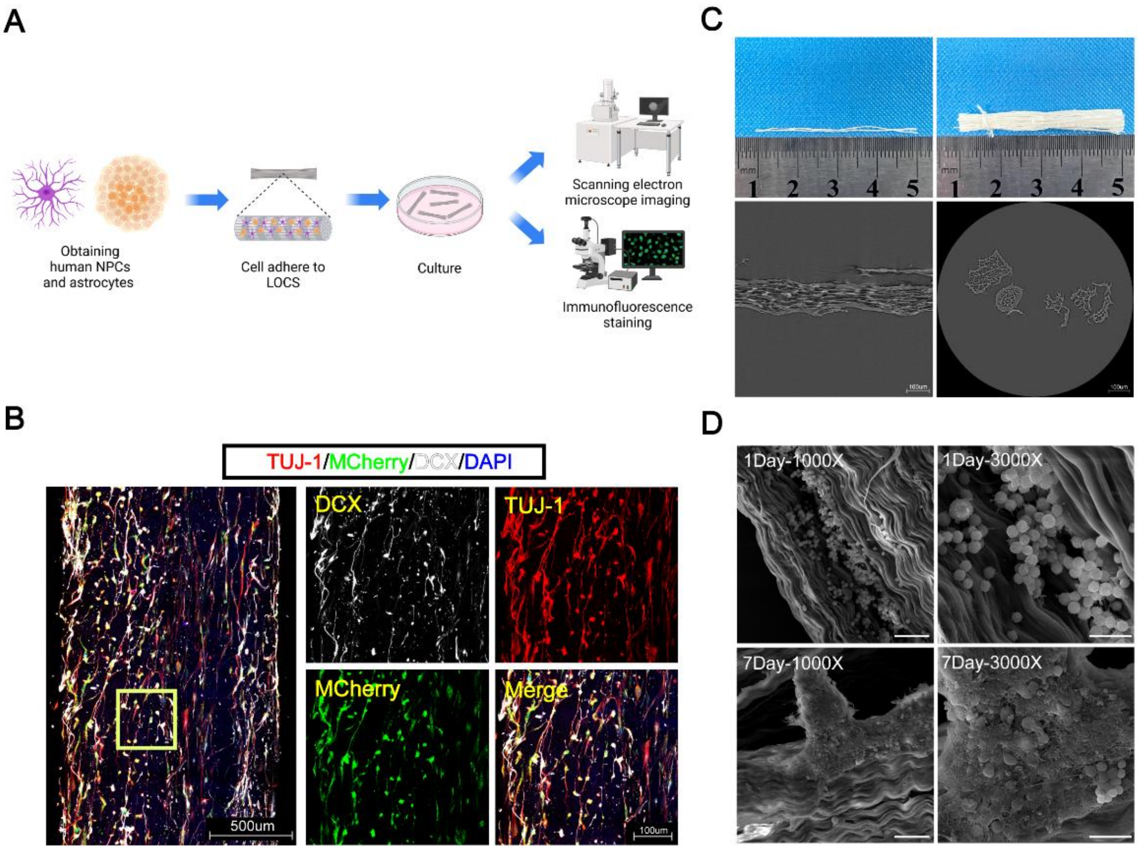

Supplementary Figures

Figure S1. The construction of hscNPCs-scaffold and characteristics. (**A**) The modification process of linearly ordered collagen scaffolds (LOCS). (**B**) In vitro neuronal differentiation of hscNPCs-scaffold after 7 days in culture. (**C**) The overviews, X-ray micrographs images of linearly ordered collagen scaffolds. (**D**) hscNPCs cultured on the collagen scaffold for 1 and 7 days. Scale bar (short)= 50 μm. Scale bar (long)= 20 μm.

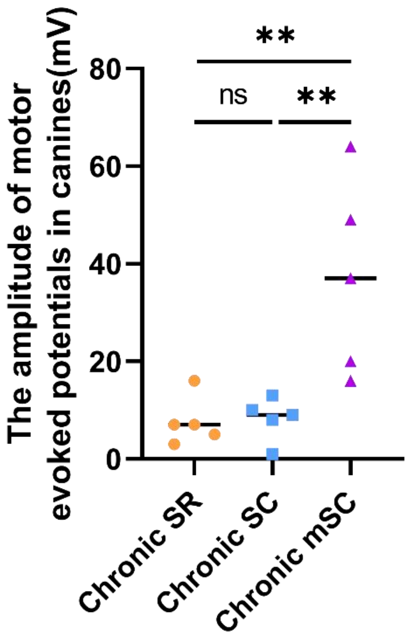

Figure S2. Electrophysiological changes after intervention. The amplitude of motor evoked potentials of three groups (n = 5). Multiple groups comparisons were analyzed using one-way ANOVA analysis of variance with Tukey's test;

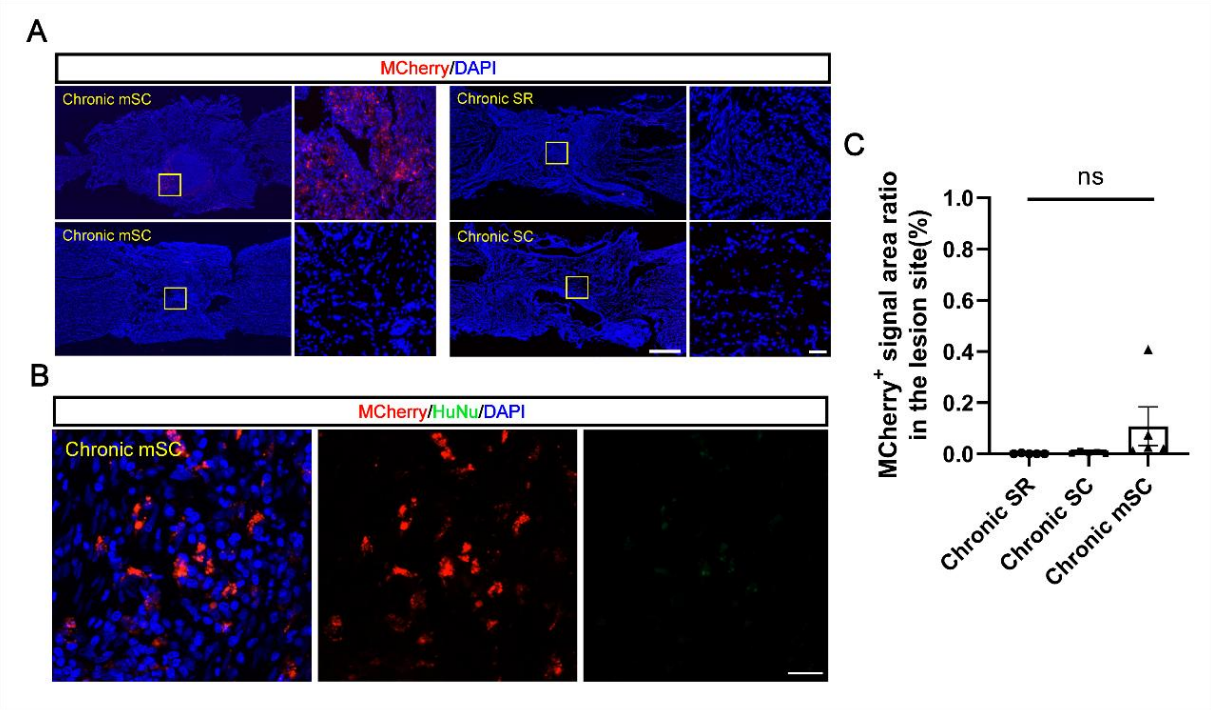

Figure S3. No graft cells survived in Chronic mSC at 5 mpi. (**A**) No MCherry$^+$ graft cell survived within the lesion area at 5mpi. Scale bar (long)= 1000 μm. Scale bar (short)= 50 μm. (**B**) Residual MCherry-positive cells do not express human nuclear antigen. Scale bar = 50 μm. (**C**) The percentage of MCherry-positive signal areas in the lesion site of three groups (n = 5). Multiple groups comparisons were analyzed using one-way ANOVA analysis of variance with Tukey's test; mean ± SEM.

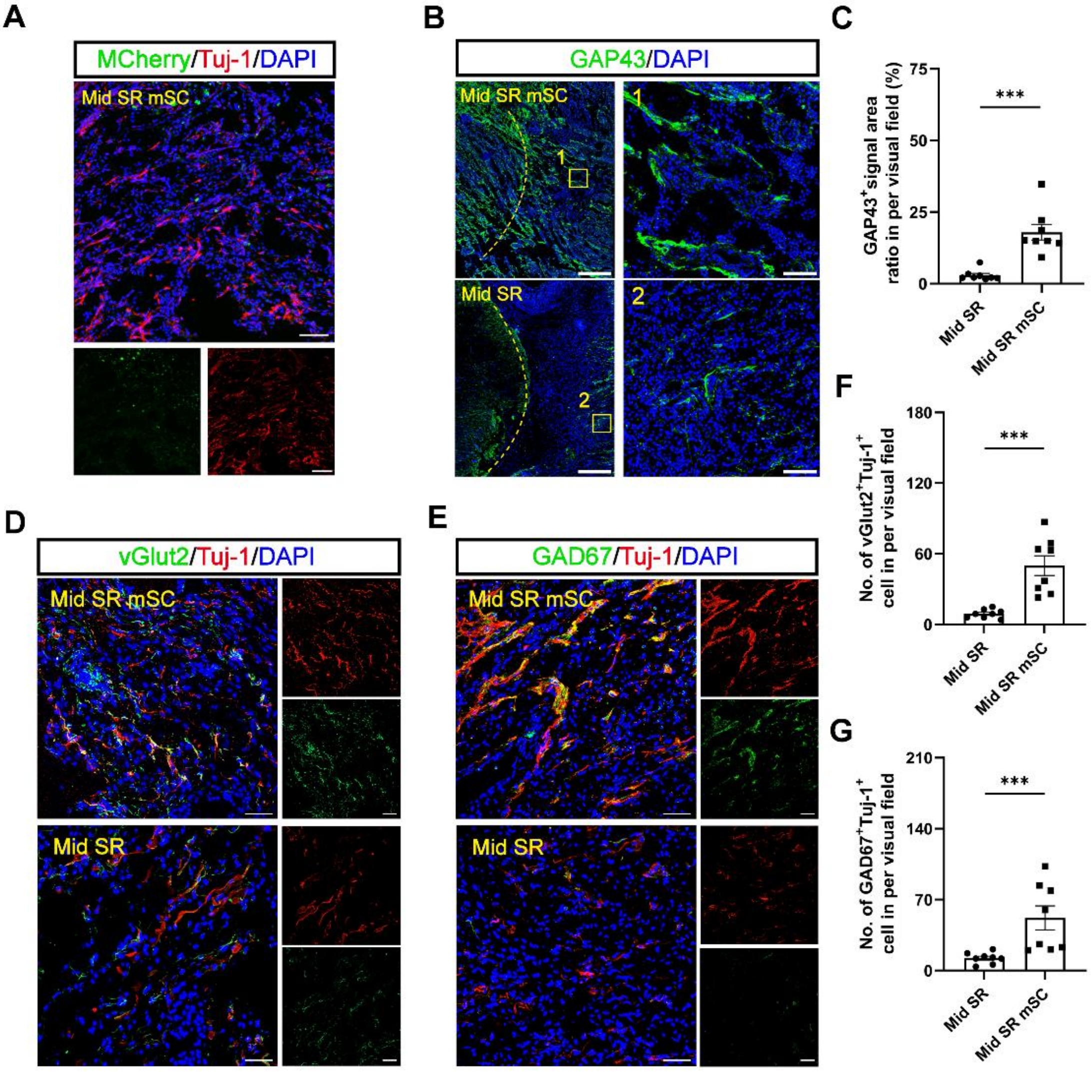

Figure S4. Endogenous functional axon regeneration between two groups. (**A**) No MCherry$^+$ graft cell co-labeled with newly regenerated Tuj-1$^+$ neurons. Scale bar = 50 μm. (**B** and **C**) GAP43$^+$ axons regenerated in the lesion site of two groups (n = 8 images). Eight images were randomly selected from the lesion site of three replicates in each group. Scale bar (long)= 500 μm. Scale bar (short)= 50 μm. (**D** to **G**) The newly formed neurons in the lesion sites of both groups expressed markers of excitatory (D) and inhibitory (E) neurons. Multiple groups comparisons were analyzed using one-way ANOVA analysis of variance with Tukey's test. $^{*}P < 0.05$, $^{**}P < 0.01$, $^{***}P < 0.001$, $^{****}P < 0.0001$; mean ± SEM.

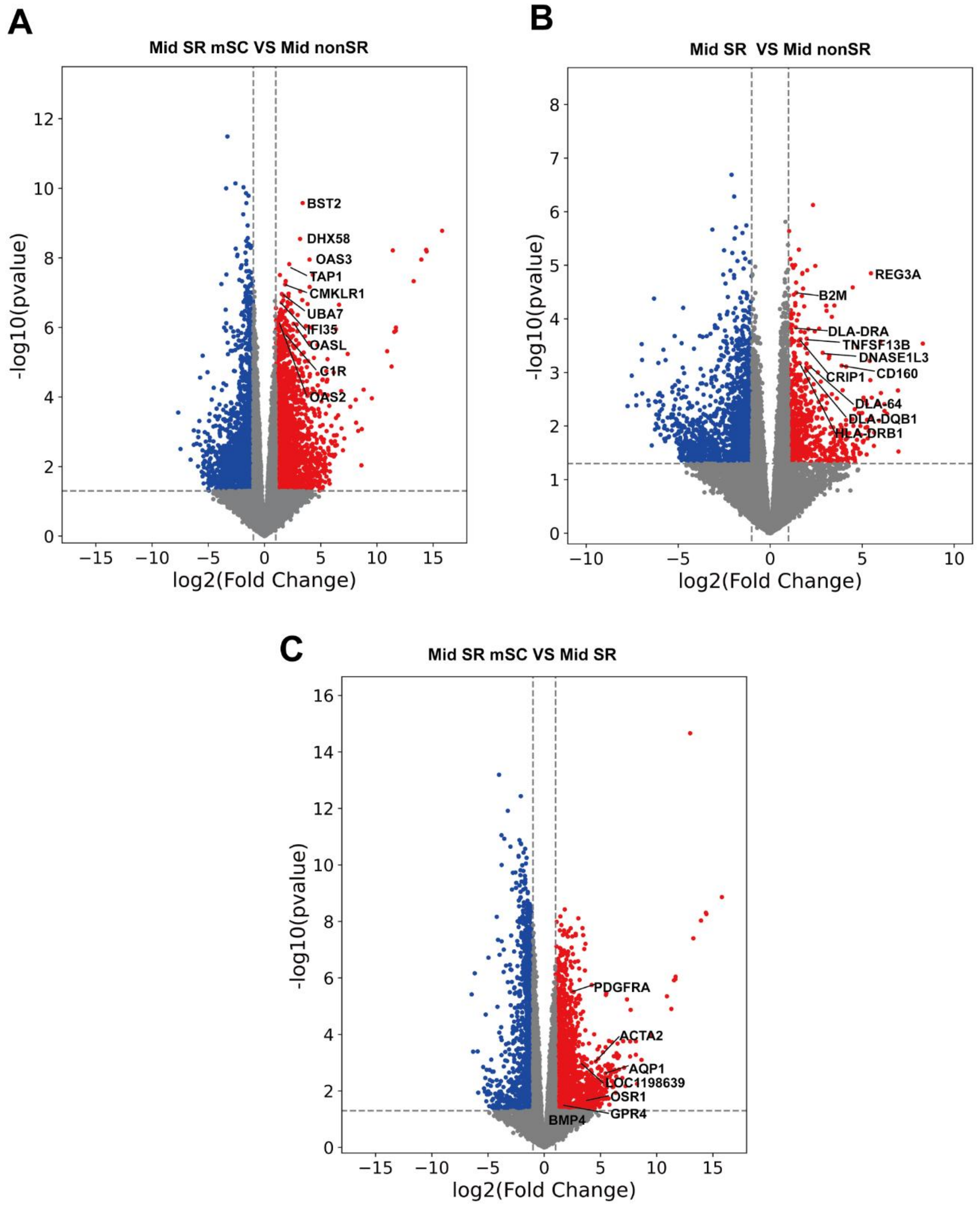

Figure S5. Genes associated with GO terms. (**A**) Top 10 upregulated genes associated with the GO term between Mid SR mSC and Mid nonSR. (**B**) Top 10 upregulated genes associated with the GO term between Mid SR and Mid nonSR. (**C**) Seven upregulated genes associated with the GO term between Mid SR mSC and Mid SR.

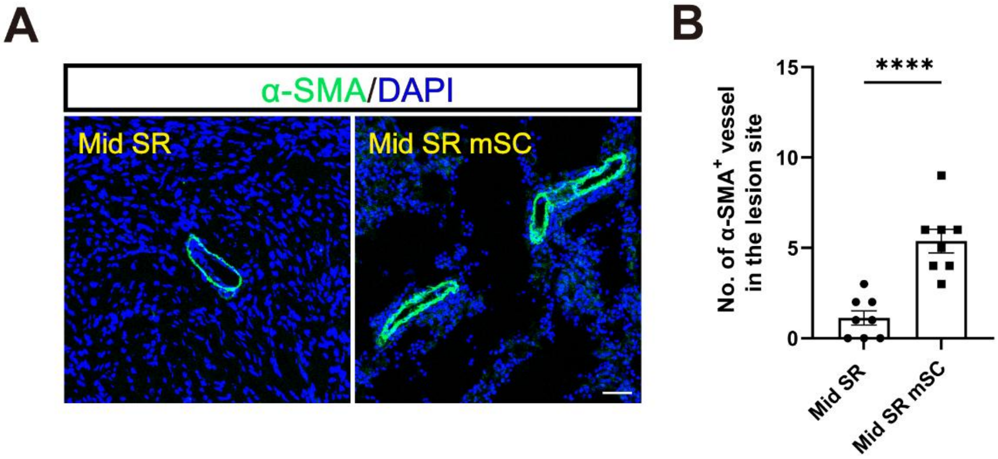

Figure S6. Immunostaining for α-SMA⁺ vessels in lesion. (**A**) Profiles of α-SMA⁺ vessels in the lesion site of two groups. Scale bar = 50 μm. (**B**) The number of α-SMA⁺ vessels in the lesion of two groups. (n = 8 images). Two-group comparisons were analyzed using unpaired t-test. **P* < 0.05; ***P* < 0.01; ****P* < 0.001; *****P* < 0.0001; mean ± SEM.

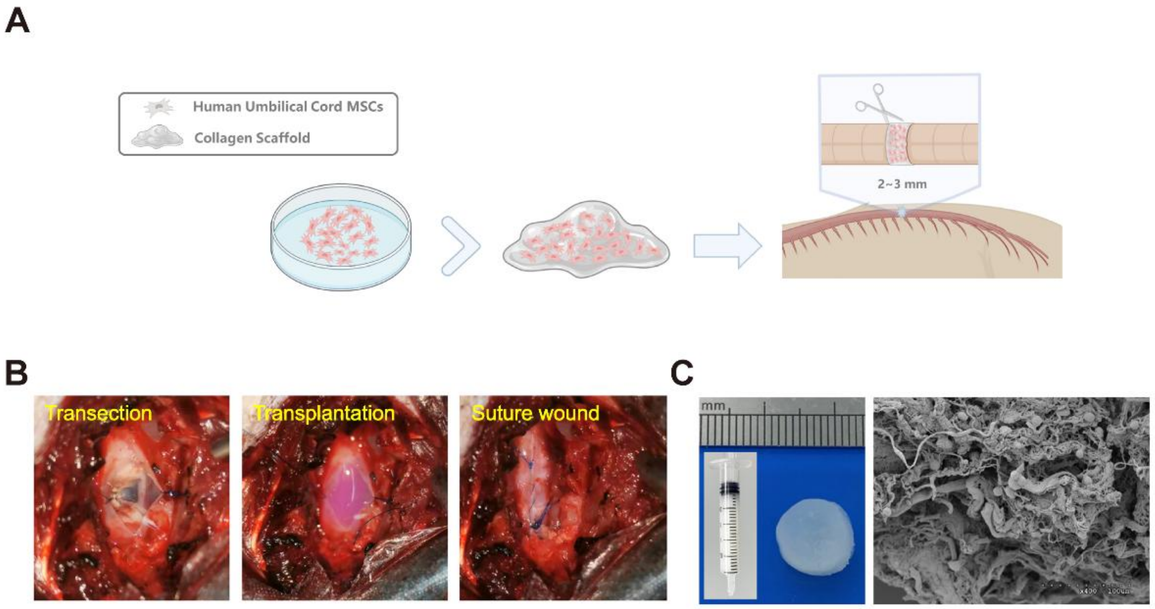

Figure S7. The process of SCI and construction of hUMSCs-scaffold. (**A**) The modification process of collagen scaffolds. (**B**) the process of SCI in rhesus monkeys. (**C**) Scaffold morphology and hUMSCs loaded scaffold shown in scanning electron microscopy image. Scale bar = 100 µm

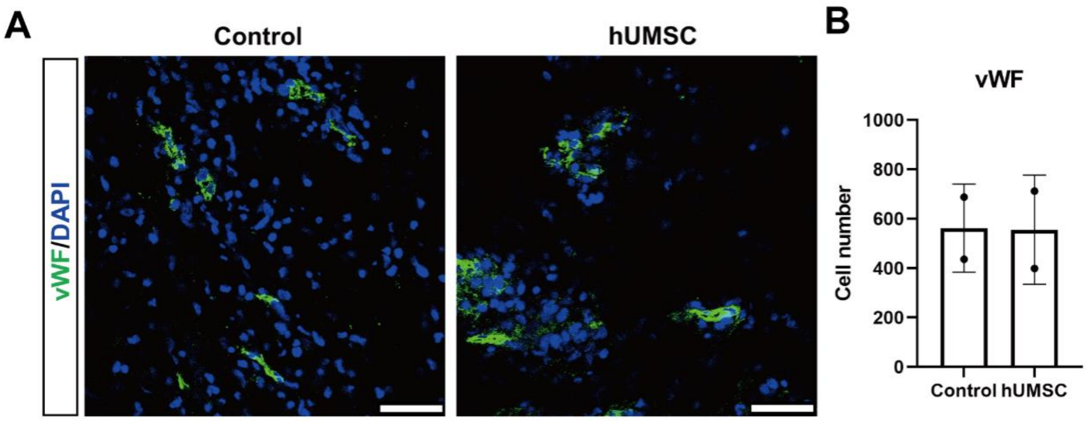

Figure S8. Survival of endothelial cells in the lesion between two groups. (**A**) vWF^+ cells within the lesion area between two groups. Scale bar = 50 µm. (**B**) The number of vWF^+ cells of lesion site between the Control and the hUMSC group; mean ± SEM.

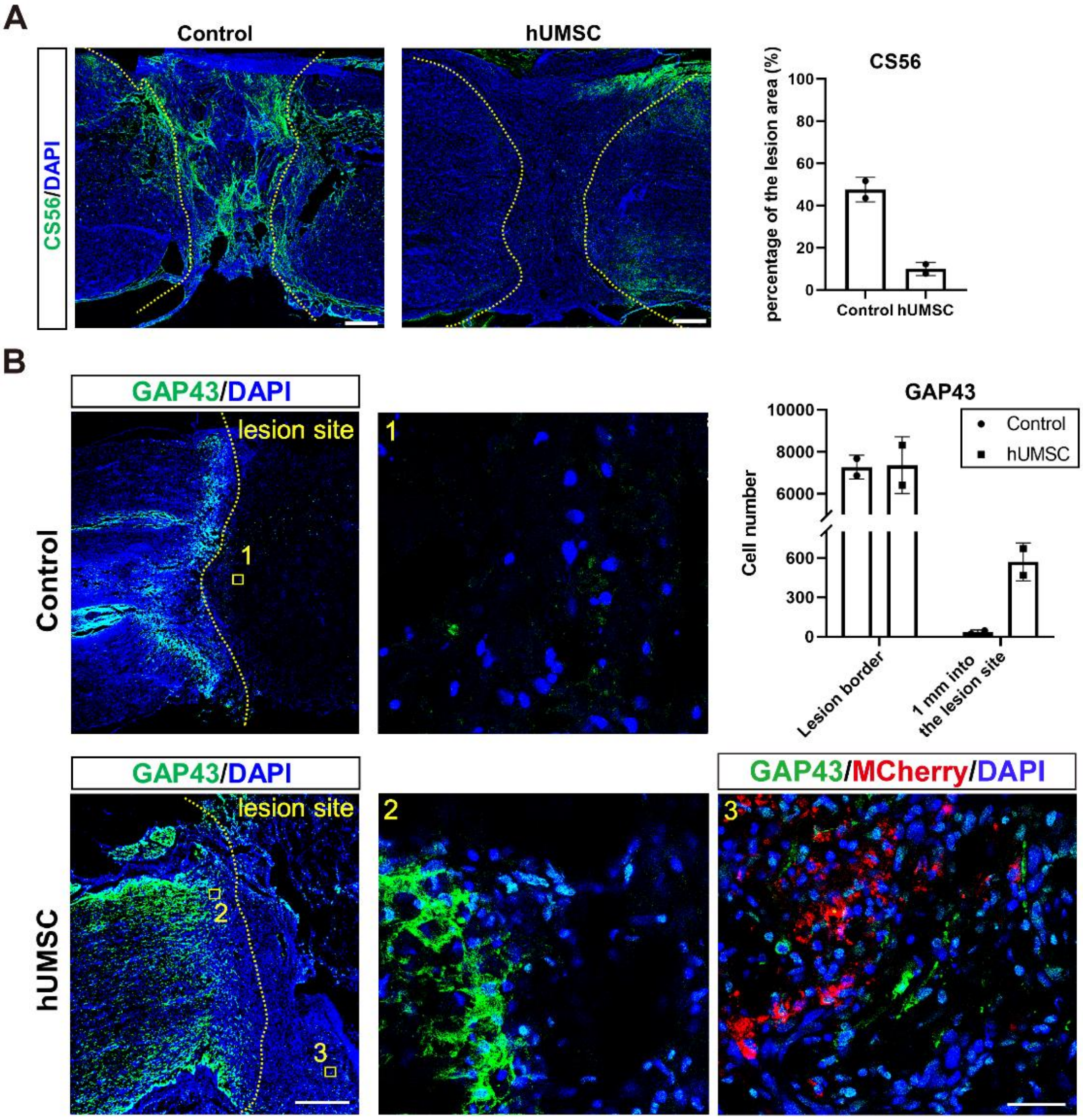

Figure S9. The accumulation of CSPGs and profile of newly regenerated axon in the lesion site. (**A**) The hUMSC group exhibited significantly less CSPG deposition compared to the control. Scale bar = 500 µm. (**B**) The central lesion area in the hUMSC group contained more endogenous newly formed axons. Scale bar (left)= 1000 µm. Scale bar (right)= 50 µm; mean ± SEM.

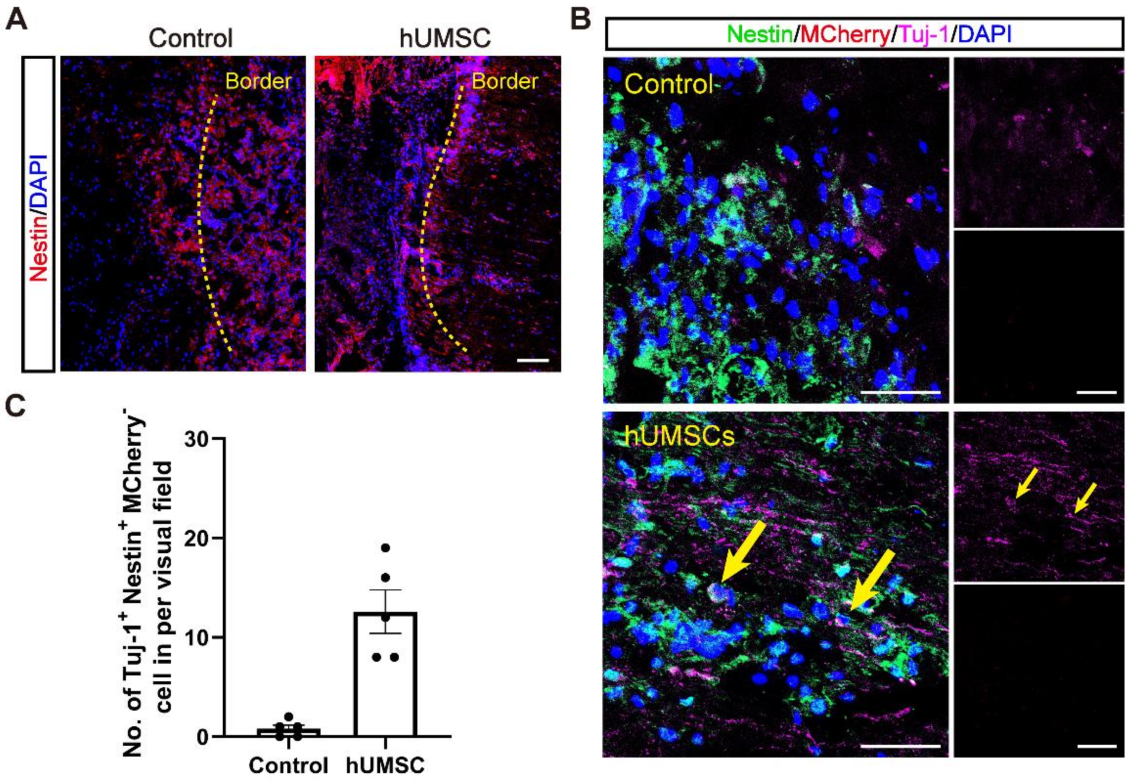

Figure S10. The neuronal differentiation and accumulation of nestin$^+$ cell in lesion of rhesus monkeys. (**A**) The accumulation of nestin$^+$ cell in lesion margins. Scale bar = 200 μm. (**B** to **C**) More nestin$^+$ cells differentiated into Tuj-1$^+$ neurons in the hUMSC group (n = 5 images). Five images were randomly selected from the lesion site of two individuals in each group. Scale bar (long)= 50 μm. Scale bar (short)= 50 μm.

Supplementary References

[51] Olby NJ, Risio LD, Muñana KR, et al. Development of a functional scoring system in dogs with acute spinal cord injuries. ajvr. 2001; 62: 1624–8.
